# A modified energy management strategy for PV/diesel hybrid system to reduce diesel consumption based on artificial protozoa optimizer

**DOI:** 10.1038/s41598-025-87716-y

**Published:** 2025-02-05

**Authors:** Rania G. Mohamed, Amal A. Hassan, Shady H. E. Abdel Aleem

**Affiliations:** 1 Department of Electrical Engineering, Institute of Aviation Engineering and Technology, Giza, 12658 Egypt; 2 Department of Photovoltaic Cells, Electronics Research Institute, Cairo, 11843 Egypt

**Keywords:** Artificial protozoa optimizer, Modified energy management strategy, Hybrid system, Photovoltaic, Diesel generators, Multi-objective optimization, Electrical and electronic engineering, Photovoltaics

## Abstract

The photovoltaic (PV)/diesel hybrid system (PV/D-HS) combines solar PV panels with a diesel generator (DG) to meet energy demands, especially in industrial operations. This study introduces an improved energy management strategy designed to optimize the performance of PV/D-HS by reducing diesel consumption, increasing solar energy utilization, and minimizing environmental impact. The strategy dynamically manages power distribution between the PV panels and the DG, adapting to changing solar conditions and energy demands. Doing so reduces the system’s reliance on diesel, improves operational efficiency, and supports the integration of cleaner energy sources. Simulation results show significant improvements over traditional approaches: carbon emissions decreased from 62 kg/day with a standalone diesel generator to 38 kg/day, representing a 38% reduction. The solar energy fraction (SEF) increases from 12 to 35%, a 23% improvement in solar energy utilization. These results demonstrate the potential of the proposed strategy to enhance sustainability by lowering greenhouse gas emissions, reducing dependence on fossil fuels, and advancing global efforts to combat climate change. In addition to environmental benefits, the approach reduces operational costs and improves the system’s reliability, making it a practical solution for industrial energy needs.

## Introduction

### Background

Hybrid energy systems (HES) combining photovoltaic (PV) power and diesel generators (DGs) have become a viable solution for providing reliable electricity in remote or off-grid areas. These systems leverage the clean, renewable energy generated by PV arrays while relying on DGs as a backup during periods of low solar availability^1–3^. However, the overuse of DGs can compromise the efficiency of such systems, leading to high operational costs, increased greenhouse gas emissions, and greater reliance on fossil fuels. Therefore, optimizing the energy management strategy (EMS) in PV diesel hybrid systems (PV/D-HSs) is critical to enhancing system efficiency and reducing diesel fuel consumption^4,5^.

### Motivation

The traditional EMSs in PV/Diesel HES often rely on simplistic control methods, such as inverter ON/OFF control, which may not fully exploit the potential of available PV energy. As a result, DGs are frequently overused, even when PV power can sufficiently meet the load demand^6,7^. The need for more sophisticated control strategies to dynamically balance power supply from PV and diesel sources while minimizing diesel usage is evident. In this context, artificial intelligence (AI) and bio-inspired optimization algorithms offer promising approaches for improving energy management in hybrid systems^8,9^. The Artificial Protozoa Optimizer (APO), a bio-inspired algorithm that mimics protozoan foraging behavior, has shown potential in optimizing complex systems^10^. This study explores the application of APO to develop a more efficient EMS with a multi-objective function for PV/D-HSs.

### Literature review

Hybrid renewable energy systems (HRES), particularly those combining PV panels and DGs, have become increasingly popular to provide reliable and sustainable power, especially in remote or off-grid areas. Integrating renewable sources like PV with traditional DGs can reduce fuel consumption, lower emissions, and enhance energy security. However, effective management of these systems is crucial to maximizing their benefits. This section reviews various management methods specifically for PV/D-HSs, discussing their strengths, limitations, and areas that require further exploration^11–15^. Rule-based control strategies (RBCS) are among the earliest and most straightforward methods for managing PV/D-HSs. These strategies operate based on predefined rules that dictate when the DG should start or stop and how the PV output should be utilized. For example, the generator may begin when the solar power is insufficient to meet the load or when the battery (if present) reaches a certain discharge level. The strengths of RBCS are simple implementation, low computational demand, ease of understanding, and deployment in various system configurations. The limitations of RBCS are, firstly, it lacks flexibility and adaptability to changing environmental conditions; secondly, it often results in suboptimal performance, with unnecessary DG operation leading to higher fuel consumption; and finally, it does not optimize the use of available renewable resources^16,17^.

Load Following (LF) and Cycle Charging (CC) are common strategies used in PV/D-HSs. In load following, the DG operates only when the load demand exceeds the power from the PV system, with the generator output matching the load. On the other hand, cycle charging involves operating the DG at its optimum efficiency, often charging batteries when excess power is available. The LF strategy can help minimize fuel consumption by using the generator only when necessary, while CC maximizes generator efficiency by maintaining constant, optimal output. However, LF may cause frequent generator starts and stops, increasing wear and maintenance costs. At the same time, CC can produce excess energy, particularly if the batteries are fully charged or if the load is low, potentially leading to fuel waste^18–20^.

Optimization-based control methods have been developed to address the limitations of rule-based and basic operational strategies in PV/D-HSs by utilizing mathematical algorithms to optimize system operation based on objectives such as minimizing fuel consumption, reducing emissions, or maximizing solar energy use. Techniques like Linear Programming (LP) and Mixed-Integer Linear Programming (MILP) are employed to optimize the DG’s operation schedule and PV power utilization. At the same time, Dynamic Programming (DP) addresses complex, time-dependent issues by breaking them into simpler sub-problems. These methods can provide near-optimal operational strategies and are flexible enough to handle multiple objectives and constraints. However, they are computationally intensive, particularly for real-time applications, and require accurate models and forecasts, which may not always be readily available^21–24^.

AI-based control methods, including fuzzy logic, neural networks, and evolutionary algorithms, have emerged as powerful tools for managing PV/D-HSs due to their ability to handle these systems’ nonlinear and complex nature and the uncertainties associated with renewable energy sources. Fuzzy logic controllers (FLC) manage the imprecise and uncertain inputs using rules based on human expertise. Neural Networks (NN) leverage historical data to predict future energy generation and consumption, enhancing real-time decision-making. Evolutionary Algorithms (EAs), such as Genetic Algorithms (GA) and Particle Swarm Optimization (PSO), are employed to optimize overall system performance by exploring a broad range of potential solutions. These AI methods are highly adaptable to changing environmental and load conditions and can optimize complex systems with conflicting objectives. However, they require extensive training data and significant computational resources, making their real-time implementation challenging due to their inherent complexity^25,26^.

Model predictive control (MPC) is an advanced strategy that forecasts future system states based on a model of the PV/D-HS and optimizes control actions over a set future time horizon. MPC is particularly effective in managing systems with multiple inputs and outputs and excels at handling operational constraints. It provides optimal control by predicting future conditions and adjusting operations accordingly, making it well-suited for multivariable systems and scenarios with multiple objectives. However, MPC comes with high computational demands, especially in large-scale or real-time applications, and its effectiveness is heavily dependent on the accuracy of the system models and the reliability of the forecasts, which may not always be readily available or accessible to obtain^27–29^.

Applying fractional-order proportional-integral-derivative (FO-PID) controllers in energy management strategies for hybrid systems, especially PV/D-HSs, has gained attention due to their enhanced control capabilities over classical PID controllers. The FO-PID controller, characterized by its fractional calculus-based control action, offers greater flexibility in tuning control parameters, improving system stability, dynamic response, and robustness^30–32^. Several studies have explored the benefits of fractional-order control in hybrid energy systems. Fractional controllers have demonstrated superior performance in handling the nonlinearities and uncertainties typical in renewable energy systems like solar power, where weather conditions introduce significant variability in power output^30–32^. The FO-PID controller has shown promise in managing the transition between renewable energy sources and backup DGs in renewable energy integration. Many studies concluded that FO-PID controllers outperformed classical PID controllers in terms of settling time and robustness in the presence of solar power intermittencies, making them suitable for energy management in PV/D-HSs^30–32^. Researchers have applied FO-PID controllers to hybrid systems to optimize power flow, especially in hybrid configurations. The fractional-order approach enabled the controller to manage better the power flow between the PV system and the DG, reducing the dependency on diesel and improving the overall system efficiency^30–32^.

In PV/D-HSs, the energy management strategy often requires optimizing multiple conflicting objectives, such as minimizing fuel consumption, reducing power supply interruptions, and maximizing the use of renewable energy. When combined with optimization algorithms, FO-PID controllers have proven effective in achieving these goals^33–35^.

In this study, the authors applied a FO-PID-based APO strategy using a multi-objective optimization approach to balance solar and diesel power, significantly reducing the net present cost (NPC) and loss of power supply probability (LPSP). These results showed that FO-PID’s flexibility in handling fractional-order derivatives allowed for more effective tuning of the energy management strategy, which led to greater energy efficiency and cost savings. In the literature review, several comparative studies have highlighted the performance improvements of FO-PID over classical PID in hybrid systems. A key advantage of FO-PID is its ability to offer non-integer control actions, which leads to better adaptability to the variable nature of renewable energy sources. FO-PID controllers showed significant improvement in handling the uncertainties of solar power in PV/D-HSs compared to traditional PID controllers, leading to fewer power supply interruptions and lower operational costs.

The combination of FO-PID control and the APO offers a novel approach to energy management that distinguishes itself from other AI-based strategies. FO-PID provides enhanced precision in control through fractional calculus, allowing more accurate tuning of system dynamics. This precise control ensures stable performance and better response to fluctuating environmental conditions and varying load demands, which is a limitation in traditional PID-based or other AI methods. APO, a bio-inspired optimization algorithm, complements FO-PID by dynamically adapting energy allocation in real-time. Mimicking the foraging behavior of protozoa, APO quickly responds to changes in solar irradiance, load demand, and fuel costs, optimizing energy usage more adaptively than static AI strategies like neural networks or genetic algorithms. This adaptability enables the system to maximize solar energy utilization while minimizing dependence on the diesel generator (DG)^36–39^. The combined FO-PID and APO strategy leverages their complementary strengths. FO-PID manages precise active power control, while APO optimizes global energy distribution, significantly improving system efficiency. The approach achieves multi-objective optimization, balancing goals such as minimizing costs, reducing carbon emissions, and lowering the LPSP. This holistic optimization outperforms AI techniques focused on single objectives or less comprehensive methods. Another unique aspect is the strategy’s ability to reduce carbon emissions significantly. The FO-PID and APO combination demonstrates superior environmental benefits by minimizing diesel consumption more effectively than other AI-based approaches. Simulation results show marked reductions in emissions compared to classical energy management strategies or standalone DG systems. Additionally, the method achieves higher solar energy utilization, showcasing its potential to enhance renewable energy integration in hybrid systems. The hybrid strategy is robust against environmental and demand fluctuations, making it highly effective in practical applications. Furthermore, its compatibility with existing hybrid energy system components facilitates real-world deployment, adding to its practicality. By integrating the precision of FO-PID with the adaptive optimization of APO, this novel approach sets a new standard for efficient, reliable, and sustainable energy management^36–39^.

### Research gap

Despite significant progress in developing and implementing management strategies for PV/D-HSs, several research gaps remain.

First, real-time optimization presents a major challenge due to the computational complexity of advanced optimization and AI-based methods. There is a pressing need for more efficient, real-time optimization algorithms that can operate within the constraints of available computational resources without compromising performance.

Second, the robustness of control methods to uncertainty remains a critical issue. Renewable energy sources like PV are inherently unpredictable due to fluctuations in solar irradiance, and current control methods often struggle to adapt to these uncertainties, leading to suboptimal system performance. Research is needed to develop resilient control strategies for these fluctuations and maintain optimal operation despite variable conditions^40,41^.

Third, while AI-based methods such as neural networks and evolutionary algorithms have been explored, there is untapped potential in integrating more advanced and hybrid AI techniques. For example, combining fuzzy logic with neural networks or integrating AI with traditional optimization techniques could result in more effective management strategies.

Fourth, scalability and flexibility are crucial as PV/D-HSs vary widely in size and configuration, from small microgrids to large power plants. Management methods must be scalable and flexible, applicable across different system sizes, and adaptable to various configurations and operational objectives^40,41^.

Fifth, existing optimization methods often focus on single objectives, such as minimizing fuel consumption or emissions. However, real-world systems typically require balancing multiple objectives, including cost, reliability, and environmental impact. There is a need for more comprehensive multi-objective optimization frameworks that can simultaneously address these conflicting goals.

Finally, long-term sustainability is often overlooked in favor of short-term operational efficiency. Future strategies should consider the system’s long-term sustainability, including maintenance and lifecycle costs of DGs and PV components and the long-term environmental impact of diesel consumption^40,41^.

While significant advancements have been made in managing PV/D-HSs, ongoing research is essential to address these gaps. Developing more efficient, adaptive, and robust management strategies will be crucial for optimizing these systems’ performance and enhancing their role in sustainable energy solutions.

APO distinguishes itself from other advanced optimization techniques, such as Genetic Algorithms (GA), Particle Swarm Optimization (PSO), and hybrid AI models, through its unique mechanism and practical benefits. APO mimics the foraging behavior of protozoa, enabling it to focus on local exploration while adaptively responding to environmental changes in real time. This makes APO particularly effective for dynamic optimization tasks, such as energy management in hybrid systems. In contrast, GA uses principles of natural selection and genetic evolution, which, while effective for global optimization, can be computationally intensive and less suited for real-time adjustments. Similarly, PSO relies on swarm intelligence to explore solution spaces but can stagnate in local optima during high-dimensional problems^34,42,43^. One of APO’s key advantages is its adaptability to real-time changes, such as fluctuating solar irradiance or varying load demands. While GA typically requires re-initialization to handle dynamic environments, and PSO can struggle with slower adaptation, APO’s dynamic optimization mechanism allows it to adjust energy allocation quickly. Hybrid AI models, which combine techniques like GA, PSO, or neural networks, can offer high adaptability but often at the expense of increased computational complexity and implementation challenges^34,42,43^. The convergence speed of APO is another notable advantage. By leveraging its adaptive local search mechanism, APO converges faster than both GA and PSO in real-time applications. GA’s convergence can vary widely depending on population size and mutation rates, often requiring numerous iterations to find optimal solutions. While generally faster than GA, PSO may still lag behind APO in dynamic scenarios. Hybrid models can occasionally outperform APO in convergence but often require significantly higher computational resources due to their combined algorithms. In terms of computational complexity, APO maintains a relatively low to moderate profile, making it efficient and practical for real-time applications. Conversely, GA involves high complexity due to operations like crossover and mutation across large populations. At the same time, PSO exhibits moderate complexity, with the computational load increasing with the number of particles and iterations. Hybrid models are typically the most complex, as integrating multiple algorithms requires additional resources and intricate parameter tuning^34,42,43^.

When applied to energy management systems, APO excels by optimizing solar energy utilization and reducing reliance on diesel generators in hybrid setups. GA, while suitable for static optimization tasks, lacks the flexibility for real-time energy management. PSO performs well in static and moderately dynamic scenarios but cannot match APO’s adaptability. Hybrid models, while highly accurate and robust, are challenging to implement in real-time systems due to their complexity and computational demands. APO effectively avoids getting trapped in local optima, a common challenge in optimization^34,42,43^.

Its adaptive movement and exploration strategies make it more robust than GA, which may lose diversity in the population and struggle with premature convergence. PSO is also prone to stagnation, especially in high-dimensional problems. Depending on their components, hybrid models can mitigate this issue, but their effectiveness varies^34,42,43^.

APO offers a unique combination of adaptability, convergence speed, computational efficiency, and ease of implementation. These strengths make it a superior choice for dynamic and real-time applications like energy management, where other advanced optimization techniques often face limitations^34,42,43^.

Table 1 compares APO with other advanced optimization techniques, such as GA, PSO, or hybrid AI models.

**Table1:** Comparison between APO and GA, PSO, hybrid AI models^34,40–43^.

Aspect	APO	GA	PSO	Hybrid Models
Mechanism	Adaptive local search	Global search with evolution	Swarm-based search	Combined strengths
Adaptability	High	Low	Moderate	Moderate to High
Convergence speed	High	Low to Moderate	Moderate	Variable
Computational complexity	Low to Moderate	High	Moderate	High
Performance in energy management	Excellent in real-time	Good for static tasks	Good, less dynamic	Excellent but resource-heavy
Avoiding local optima	High	Moderate	Moderate	High
Ease of implementation	Easy	Moderate	Moderate	Complex

The APO stands out for its real-time adaptability, computational efficiency, and simplicity, making it well-suited for dynamic energy management systems.

In this work, the proposed MEMS, which utilizes the FO-PID controller and the Adaptive Population Optimization (APO) algorithm, addresses several key challenges typically faced by hybrid energy systems. One of the main challenges in hybrid PV/D-HSs is achieving real-time optimization under dynamic environmental conditions. The FO-PID-based APO approach ensures dynamic adjustments to the inverter’s active power output, enabling the system to adapt rapidly to fluctuating solar irradiance, load demands, and system conditions. This dynamic adjustment is critical in real-time optimization, particularly in systems with varying renewable energy contributions and load profiles.

Regarding scalability, MEMS offers a flexible solution by optimizing the balance between power contributions from the PV array and the diesel generator (DG) without requiring significant computational resources. The APO algorithm’s adaptive nature ensures it can operate efficiently across systems of varying sizes. By maintaining low to moderate computational complexity, the system remains scalable to larger hybrid energy configurations while ensuring that performance does not degrade as system complexity increases. Regarding environmental variability, MEMS addresses the challenge of the unpredictability of renewable energy, particularly solar power. With fractional-order control capabilities, the FO-PID controller enables precise tuning of system parameters, enhancing its robustness to fluctuating weather conditions and solar irradiance. The APO algorithm, mimicking the foraging behavior of protozoa, further enhances system adaptability by allowing quick responses to dynamic environmental changes, reducing reliance on diesel generators, and maximizing PV power utilization. The integration of energy storage is another challenge faced by many hybrid systems. While primarily focused on PV and DG integration, MEMS also accounts for energy storage systems within its optimization framework. By dynamically adjusting the active power output, MEMS ensures that energy storage systems are utilized efficiently, thus reducing excess energy wastage and maximizing stored energy utilization when required. This energy management approach effectively balances power generation and storage cycles. For long-term performance and sustainability, MEMS addresses uncertainties in renewable energy production and load demands. The combination of FO-PID’s adaptability and APO’s global optimization capabilities allows for effective forecasting and balancing of energy generation and consumption. The approach reduces operational costs by minimizing fuel consumption and optimizing energy storage, thus contributing to a more sustainable long-term operation. Furthermore, incorporating the FO-PID controller and APO algorithm helps overcome the challenge of minimizing fuel consumption and reducing the environmental impact, a key limitation in traditional EMS strategies. By optimizing the energy flow and ensuring that PV power is maximized while minimizing the DG’s operational hours, MEMS effectively reduces fuel usage and lowers carbon emissions, contributing to both operational efficiency and environmental sustainability. The MEMS framework, with its advanced combination of FO-PID control and the APO algorithm, offers an innovative solution to the key challenges of real-time optimization, environmental adaptability, scalability, and energy storage management. Through dynamic, real-time optimization and the reduction of fuel consumption, MEMS enhances the efficiency and sustainability of hybrid energy systems, making it a promising solution for improving the performance of PV/D-HSs.

### Contributions

The main contributions of the study are given as follows:

- Introduces a modified energy management strategy (MEMS) for a PV/D-HS tailored for an industrial chemical production facility in Qalyubia Governorate, Egypt, to enhance energy efficiency and reduce operational costs.
- Implements the FO-PID controller combined with the APO to minimize conflicting objectives such as LPSP and NPC simultaneously.
- The multi-objective optimization tool APO algorithm minimizes diesel fuel consumption and maintenance costs while maximizing solar energy (PV power) utilization.
- This paper introduces a comparison between a classical energy management strategy (CEMS) based on inverter ON/OFF control (IOOC) and a MEMS-based on inverter active power control (IAPC) using FO-PID and APO.
- MATLAB simulation results significantly improved energy system performance, reducing the LPSP and NPC and ensuring more reliable power for industrial operations.
- The simulation further revealed reduced greenhouse gas emissions, as the system maximized PV power utilization and minimized reliance on DGs.

### Paper organization

The following sections comprise the paper’s division: The introduction and literature review are presented in Part 1. Part 2 also includes the case that is being studied. Furthermore, Part 3 provides mathematical models of the hybrid PV/diesel system component. The APO is explained in Part 4. Moreover, energy management techniques for hybrid PV/diesel energy systems are presented in Part 5. Part 6 also includes a discussion, comparative statistical analysis, and the simulation findings. Part 7 points to the conclusions.

## Case under study

**Fig. 1:**
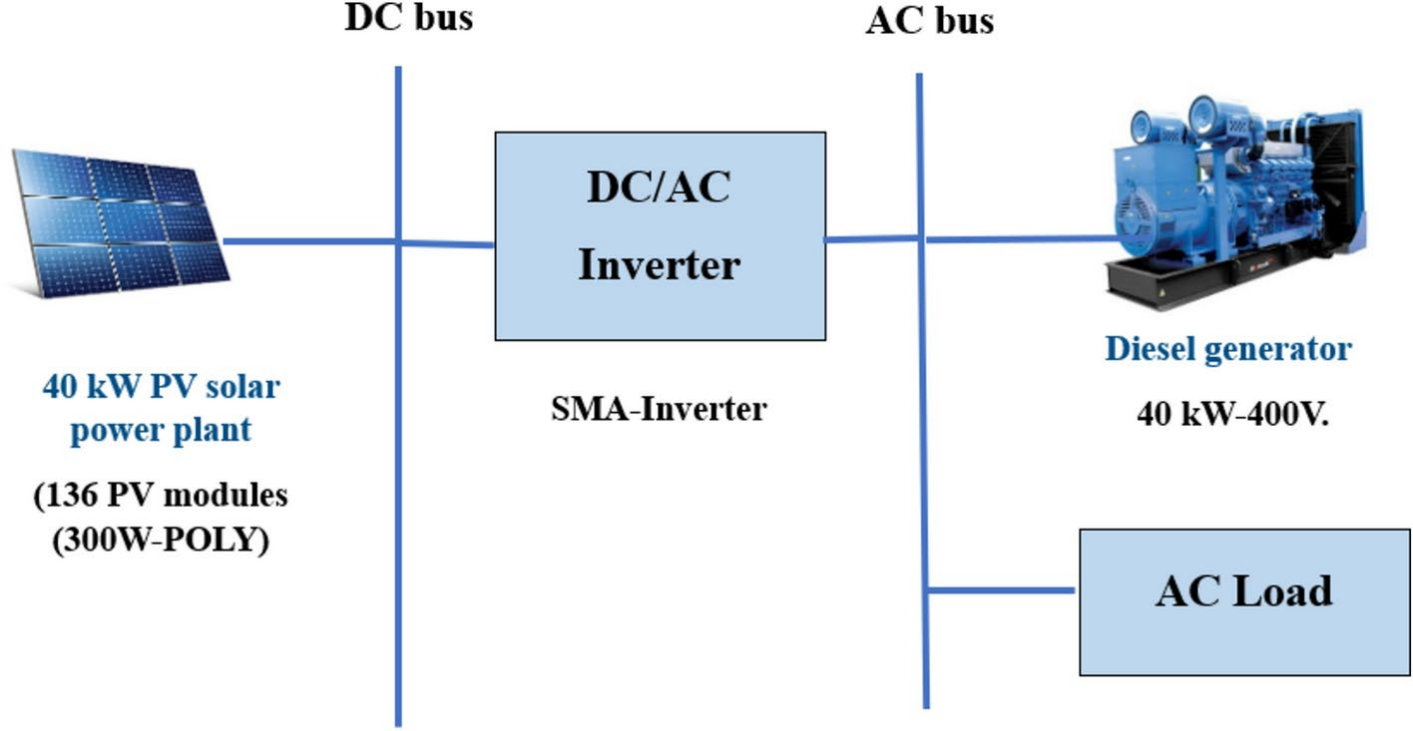
Configuration of the PV/D-HS at the factory.

This work was conducted at an industrial factory for Chemicals production in Qalyubia Governorate-Egypt. Factory, a major industrial player, requires a reliable and efficient energy supply to maintain its operations. The factory has implemented an HES that combines PV solar power and DGs to meet its energy demands. This hybrid system is crucial in ensuring uninterrupted power supply while aiming to reduce operational costs and environmental impact. HES consists of a solar power plant with a capacity of 40 kW and one DG 40 kW-400V. Figure 1 shows PV/D-HS at the factory.

As depicted in Fig. 1, the PV system harnesses solar energy to generate electricity. It operates primarily during daylight hours, contributing renewable energy to the power mix. The goal is to maximize the use of solar power to reduce reliance on DGs, thereby lowering fuel consumption and emissions. The DGs provide the necessary backup power, ensuring a continuous energy supply when solar power is insufficient (e.g., during nighttime or cloudy days). These generators are also critical during periods of peak demand, where the PV system alone may not meet the energy requirements.

Table 2 presents the technical specifications of a hybrid PV and diesel generator (D-HS) system, which integrates PV arrays, a diesel generator, and an inverter to generate and manage energy. The PV array has a nominal maximum power of 300 W, with a maximum power voltage of 37.02 V and a maximum power current of 8.11 A. It operates efficiently at 15.5%, with an open circuit voltage of 44.52 V and a short circuit current of 9.06 A, and can handle temperatures ranging from −40 °C to + 85 °C. The diesel generator is rated at 40 kW, with a minimum load ratio of 10 kW, consuming fuel at a rate of 2.73 L/hr at output and 1.32 L/hr at rated power. Emissions from the diesel generator include 7153.7 g/hr of CO2, 44.6 g/hr of CO, 18.0 g/hr of NOx, and 54.6 g/hr of SO2. The inverter, with a rated output of 40 kW, has an efficiency of 98% and operates within an input voltage range of 300 VDC to 800 VDC. It outputs 400 VAC with a frequency of 50/60 Hz, supporting a current of 75 A on the input and 60 A on the output. The inverter’s power factor is unity (1.0), with a total harmonic distortion (THD) under 3%, and features forced air cooling with protection against overvoltage, overcurrent, short circuits, and thermal conditions, making it suitable for outdoor use with an operating temperature range of −20 °C to + 60 °C.

**Table 2:** Technical specifications of PV/D-HS System^34,40–43^.

Component	Technical specifications		
**PV array**	**Nominal max power**	300 W	
	**Maximum power voltage**	37.02 V	
	**Maximum power current**	8.11 A	
	**Open circuit voltage**	44.52 V	
	**Short circuit current**	9.06 A	
	**Efficiency**	15.50%	
	**Operating temperature**	−40 + 85 (^o^C)	
	**Transmittance**	0.9	
	**Temperature Coefficient**	−0.346%/℃	
**Diesel** **Generator**	**Rated power**	40 kW	
	**Minimum load ratio**	10 kW	
	**Diesel generator** **fuel curve:**	**a / output**	2.73 L/hr
		**b/ rated**	1.32 L/hr
	**Emissions produced by Diesel generator(g/L)**	**CO**_**2**_	7153.7 g/hr
		**CO**	44.6 g/hr
		**NOx**	18.0 g/hr
		**SO**_**2**_	54.6 g/hr
**Inverter**	**Rated power output**	40 kW	
	**Efficiency**	98%	
	**Input voltage range**	300 VDC to 800 VDC	
	**Output voltage**	400 VAC (three-phase)	
	**Frequency**	50/60 Hz	
	**Input current**	75 A (at rated power)	
	**Output current**	60 A (at rated power)	
	**Power factor**	1.0 (unity)	
	**THD**	< 3%	
	**Cooling type**	Forced air cooling	
	**Operating temperature range**	−20 °C to + 60 °C	
	**Protection**	Overvoltage, overcurrent, short-circuit, thermal, islanding	

### Load profile

**Fig. 2:**
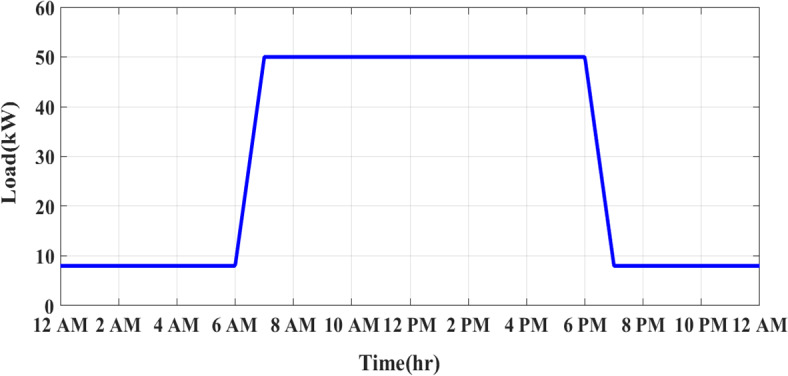
Load profile at the factory.

The load profile represents the variation in electricity demand over time and helps optimize the use of solar energy while minimizing diesel consumption. The factory’s energy demand consists of a peak load of 50 kW during working hours (6 AM—6 PM) and a reduced load of 8 kW during off-hours (6 PM—5 AM). Figure 2 shows the load profile at the factory.

### Solar radiation and temperature

Qalyubia Governorate, located in northern Egypt near Cairo, benefits from favorable solar radiation levels and moderate temperature conditions, making it suitable for solar energy projects. Qalyubia experiences high solar radiation levels due to Egypt’s general climate, characterized by long sunny days and minimal cloud cover.

The region receives an average of 5.5 to 6.5 kWh/m^2^/day of solar energy, which is typical for most areas in Egypt. This high solar potential allows for efficient solar power generation, especially during summer when sunlight is most robust and consistent. The average number of sunshine hours per day ranges from 8 to 10 h^44^.

Also, Qalyubia experiences a Mediterranean climate with hot summers and mild winters. Summer temperatures can reach 35°C to 40°C (95°F to 104°F), while winter temperatures range from 10°C to 20°C (50°F to 68°F).

**Fig. 3:**
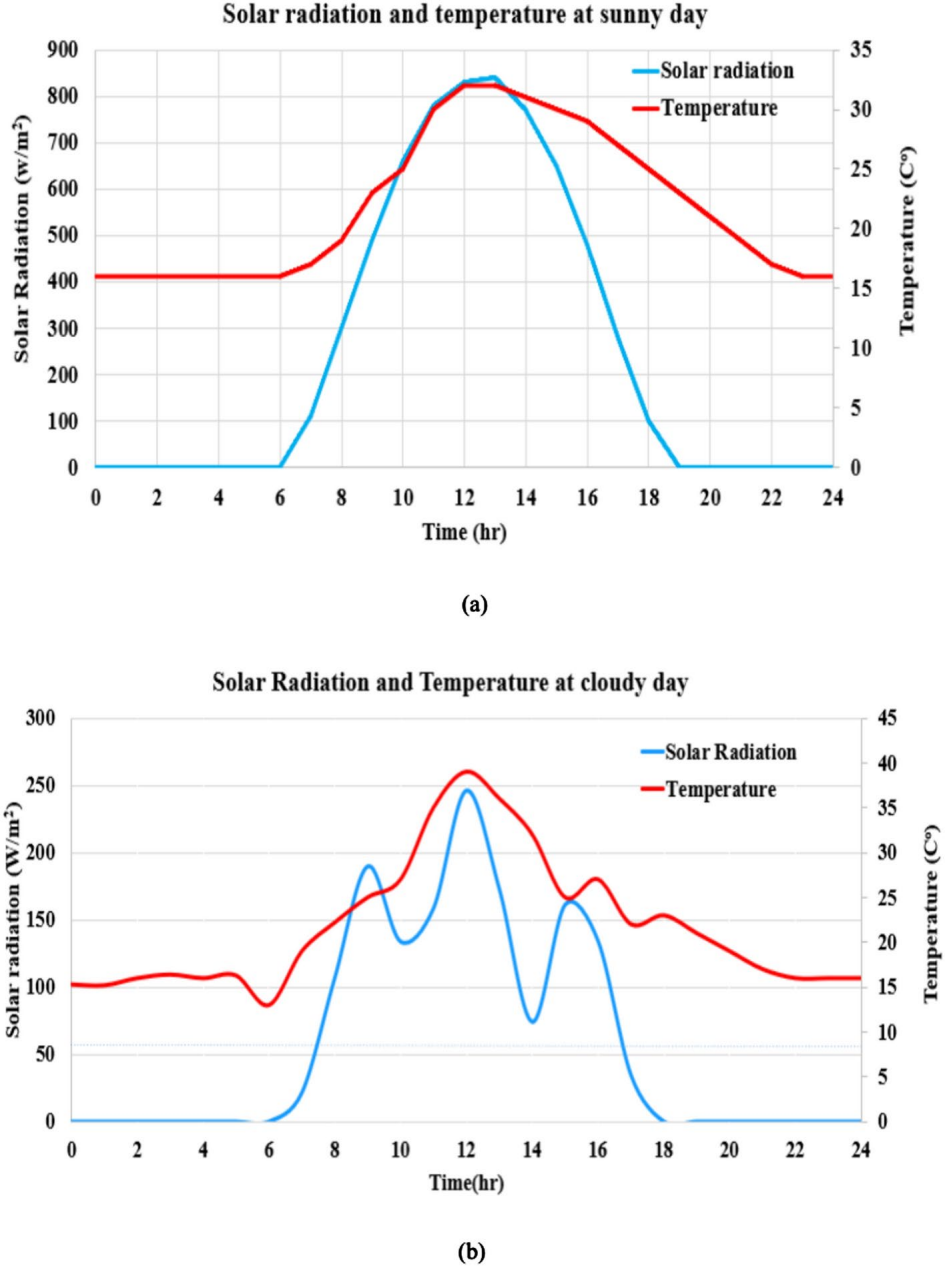
Realistic waveforms of solar radiation and temperature at Qalyubia City, Egypt. (**a**) Sunny day, and (**b**) cloudy day.

Though high summer temperatures can slightly reduce the efficiency of PV solar panels due to the heat, the region’s overall solar potential remains strong. With proper system design and cooling mechanisms, these effects can be mitigated. Solar radiation and temperature data on sunny and cloudy days specific to Qalyubia City, Egypt, are shown in Fig. 3^44^.

## Mathematical models of hybrid PV/diesel system components

### Mathematical model of the PV system

Solar irradiance and temperature influence the PV system’s output power, which is shown in Eq. (1)^36,38,39,45^.

1 $${P}_{PV}\left(t\right)={\eta }_{PV}\times {A}_{PV}\times G\left(t\right)\times \left[1-{\alpha }_{T}\times \left({T}_{c}\left(t\right)-{T}_{ref}\right)\right]$$

${P}_{PV}\left(t\right)$: Power output of the PV array at time $t$ (kW).

${\eta }_{PV}$: Efficiency of the PV modules (%).

${A}_{PV}$: Area of the PV array (m^2^).

$G(t)$: Solar irradiance on the PV array at $t$ (kW/m^2^).

${\alpha }_{T}$: Temperature coefficient of power (1/°C).

${T}_{c}\left(t\right)$: Cell temperature at time $t$ (°C).

${T}_{ref}$: Reference temperature (usually 25°C).

The PV cell temperature is shown in Eq. (2)^36,38,39,45^.

2 $${T}_{c}\left(t\right)={T}_{a}\left(t\right)+\frac{G(t)}{{G}_{NOCT}} \times \left({T}_{NOCT}-{T}_{a}\right)$$

${T}_{a}\left(t\right)$: Ambient temperature at time $t$ (°C).

${T}_{NOCT}$: Nominal Operating Cell Temperature (°C).

${G}_{NOCT}$: Irradiance at NOCT (typically 0.8 kW/m^2^).

### Mathematical model of DG

The DG’s role is to supply power when the PV system’s output is insufficient to meet the load. DG power output and fuel consumption are shown in Eq. (3) and Eq. (4), respectively^36,38,39,45^.

3 $${P}_{DG}\left(t\right)={P}_{load}\left(t\right)-{P}_{PV}\left(t\right)$$

${P}_{DG}\left(t\right)$: Power output of the DG at time $t$(kW).

${P}_{load}\left(t\right)$: Power demand/load at time $t$ (kW).

${P}_{PV}\left(t\right)$: Power output of the PV array at time $t$ (kW)

4 $${F}_{DG}\left(t\right)={a \times P}_{DG}\left(t\right)+b\times {P}_{DG}{\left(t\right)}^{2}$$

${F}_{DG}\left(t\right)$: Fuel consumption rate at time t (liters/hour or kg/hour), and $a ,b$ are the fuel consumption coefficients (constants depending on the generator).

### Mathematical model of inverter

The inverter’s role is crucial as it converts the DC output from PV panels into AC and ensures seamless operation with DGs, which often supply supplementary power. The efficiency of the inverter ($\eta$). This critical parameter determines how effectively it converts DC power to AC power. It is defined as the ratio of the output AC power ​${P}_{AC\_out}\left(t\right)$ to the input DC power ${P}_{DC\_in }\left(t\right)$:

5 $${\eta }_{INV}=\frac{{P}_{AC\_out}\left(t\right)}{{P}_{DC\_in }\left(t\right)}$$

The actual AC power output can then be calculated by multiplying the DC power input by the efficiency:

6 $${P}_{AC\_out}\left(t\right)={\eta }_{INV} . {P}_{DC\_in }\left(t\right)$$

Equation (5) highlights the importance of inverter efficiency in maximizing the usable AC power from the DC source, directly affecting the overall performance of the energy system. In this work, inverter efficiency is 98%^36,38,39,45^. Inverters in hybrid systems employ control strategies to regulate power output and manage the interaction between PV and DG outputs. These control strategies typically involve dynamic equations that govern how the inverter responds to changes in power demand and input conditions. For instance, Maximum Power Point Tracking (MPPT) algorithms are used to optimize the PV output by adjusting the inverter’s operating point to match the peak power output of the solar panels^36,38,39,45^.

## Artificial protozoa optimizer (APO)

The Artificial Protozoa Optimizer (APO) is a relatively recent optimization algorithm inspired by the behavior and movement of protozoa, particularly their foraging and hunting strategies. Protozoa are single-celled organisms that move in response to various environmental stimuli, searching for nutrients or prey. The APO algorithm mimics these natural behaviors to solve optimization problems, typically in complex, multi-dimensional search spaces. Protozoa move in their environment, allowing them to explore (search for nutrients) and exploit (consume nutrients). In the context of APO, this movement is represented by updating the position of candidate solutions in the search space. The movement of protozoa can be mathematically modeled by considering a balance between exploration and exploitation, typically through a velocity vector that determines the direction and magnitude of movement^10,46^. The position of a protozoa (i.e., a candidate solution) at time $t+1$ can be updated using the following equation:

7 $${X}_{i}\left(t+1\right)= {X}_{i}\left(t\right)+velocity vecto$$

where, ${X}_{i}\left(t\right)$ represents the position of the $i$ th protozoa at $t$, and the velocity vector determines how the position changes based on environmental stimuli (nutrient gradients or the presence of predators)^10,46^.

The velocity vector can be expressed as:

8 $${V}_{i}\left(t+1\right)= {w.V}_{i}\left(t\right)+ {C}_{1}.{r}_{1}.\left({P}_{{best}_{i}}-{X}_{i}\left(t\right)\right)+{C}_{2}.{r}_{2}.\left({G}_{best}-{X}_{i}\right)$$

where, ${V}_{i}\left(t\right) :$ The velocity of protozoa $i$ at time $t$. ω: the inertia weight, balancing exploration and exploitation. ${C}_{1}$​ and ${C}_{2} :$ are cognitive and social coefficients that determine the influence of personal best position ${P}_{{best}_{i}}$ and global best position ${G}_{best}$ ​, respectively. ${r}_{1}$ and ${r}_{2}$: are random numbers uniformly distributed in the range [0, 1], adding stochasticity to the movement. The APO algorithm incorporates the foraging behavior by adjusting the velocity and position of protozoa based on nutrient concentration. Areas with high nutrient concentrations (analogous to optimal solutions) attract protozoa, while areas with low concentrations are avoided^10,46^. Protozoa can detect and respond to environmental stimuli such as nutrient concentration gradients. This can be modeled using a fitness function that evaluates how “good” a solution is, with higher fitness indicating more nutrients. The algorithm iteratively updates protozoa positions to converge toward the best solutions in the search space. The APO algorithm iteratively updates protozoa positions until a convergence criterion is met, such as a predefined number of iterations or a sufficiently small change in the best solution’s fitness^10,46^. An enhanced position update equation might include additional terms to simulate nutrient gradients and protozoa interactions^10,46^:

Given a protozoon $i$ at time $t$, its new position ${X}_{i}\left(t+1\right)$ might be updated as follows:

9 $${X}_{i}\left(t+1\right)= {X}_{i}\left(t\right)+{V}_{i}\left(t\right)+\alpha .Gradient \left({X}_{i}\left(t\right)\right)+\beta .Interaction\left({X}_{i}\left(t\right),{X}_{ij}\left(t\right)\right)$$

$\alpha$ : is a learning factor for the gradient of the nutrient field. $Gradient \left({X}_{i}\left(t\right)\right)$ : represents the local nutrient gradient. $\beta$ : is a coefficient representing interaction with other protozoa $i$.

Equation (9) illustrates the balance between exploration (through velocity and nutrient gradient) and exploitation (through interaction and movement toward better solutions)^10,46^. Protozoa evaluate the quality of their current position using a fitness function, which simulates their response to environmental stimuli (e.g., nutrient concentration)^10,46^:

10 $$\text{Fitness }\left({X}_{i}\left(t\right)\right)= f\left({X}_{i}\left(t\right)\right) 1$$

$\text{f}\left({X}_{i}\left(t\right)\right)$ represents the fitness function evaluating the quality of the position ${X}_{i}\left(t\right)$, where higher fitness corresponds to better solutions (analogous to higher nutrient concentrations)^10,46^. In some cases, the velocity may be constrained to prevent the protozoa from moving too fast:

11 $${V}_{max}\ge {V}_{i}\left(t+1\right) \ge {V}_{min}$$

**Fig. 4:**
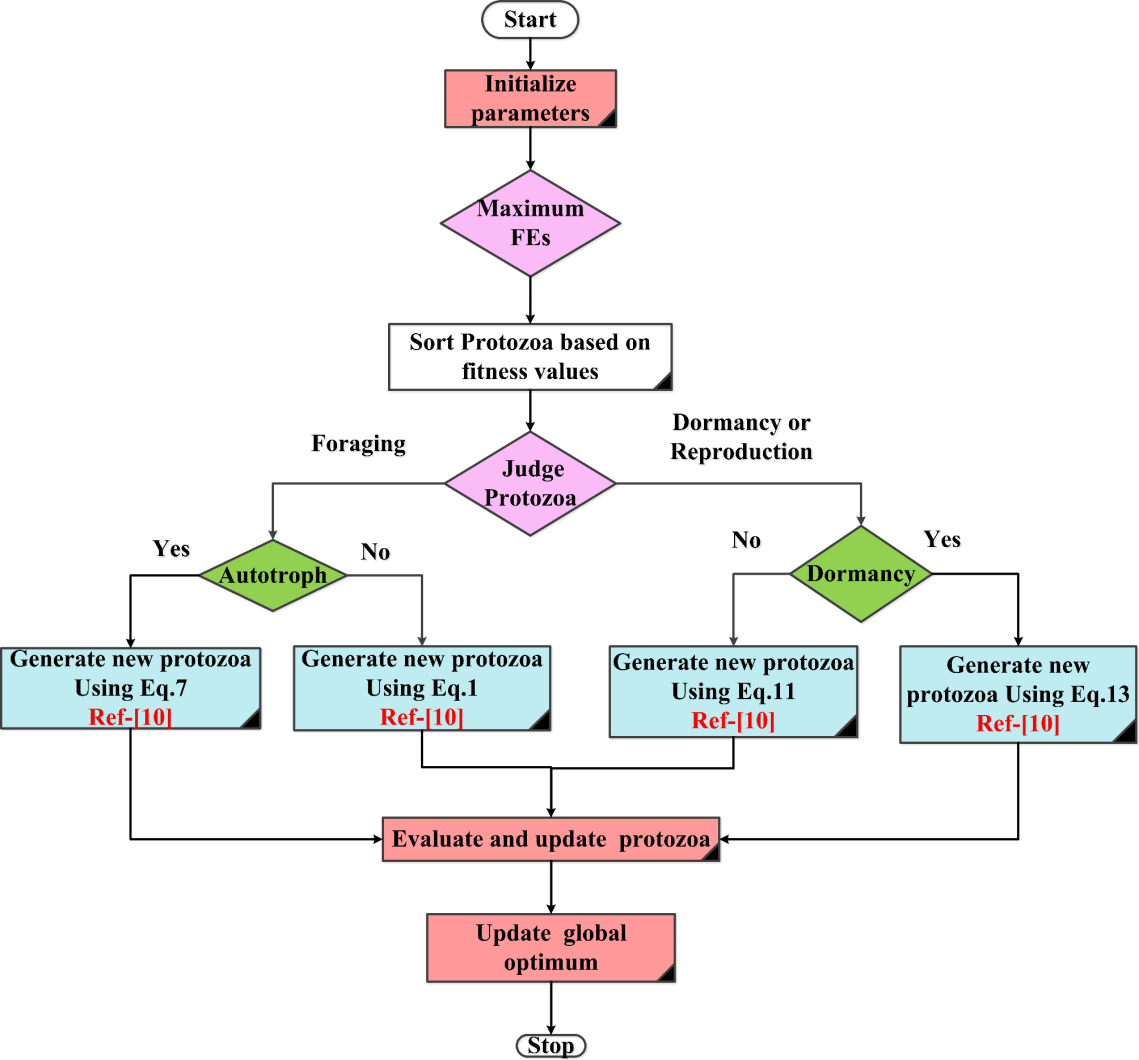
The framework of the APO.

${V}_{max}$​ and ${V}_{min}$​: Upper and lower bounds on the velocity, respectively. Figure 4shows the framework of the proposed algorithm. Traditional optimization methods may struggle with the multi-objective nature of energy management in hybrid systems, such as minimizing fuel consumption, reducing operational costs, and maximizing the utilization of renewable energy. APO’s unique approach to exploring the search space allows it to balance these competing objectives effectively, providing a more robust and flexible solution than conventional techniques^46^. APO is designed to find near-optimal solutions efficiently without requiring excessive computational resources. Its flexibility in adapting to various constraints and conditions makes it particularly useful in real-time energy management scenarios, where quick and accurate decision-making is critical. The behavior of protozoa in the APO algorithm mirrors the ability of living organisms to adapt to changing environments. This characteristic is crucial in energy management systems that must respond to fluctuating solar energy availability, varying load demands, and changes in fuel costs. APO’s adaptive mechanism allows it to optimize energy distribution in response to these changes, ensuring consistent and reliable system performance^10,46^.

Energy management in hybrid systems involves uncertainties such as unpredictable weather patterns affecting PV output and varying fuel prices. APO’s probabilistic and heuristic nature enables it to effectively handle these uncertainties, making it a robust tool for optimizing energy management under variable conditions. Compared to other optimization algorithms, APO has shown better convergence properties and solution quality in various applications. This is particularly important in HESs, where finding a global optimum or near-optimum is critical for minimizing costs and maximizing efficiency^47–50^. APO’s ability to avoid local minima and explore a vast search space contributes to finding superior solutions. As HESs grow in scale, with more components and extensive geographic spread, the need for scalable optimization algorithms becomes more pressing. APO’s computational efficiency and ability to handle large problem spaces make it a viable option for large-scale PV/D-HSs, ensuring that optimization remains effective even as the system size increases. As the energy sector evolves, integrating advanced technologies like smart grids, demand response, and the Internet of Things (IoT) enabled energy management systems becomes essential^46^. APO’s adaptability and compatibility with these emerging technologies make it a forward-looking choice, capable of evolving alongside the energy landscape. The motivation for using APO in the energy management of a PV/Diesel PV/D-HS lies in its ability to handle the system’s complexity, adapt to changing conditions, and optimize multiple competing objectives efficiently. Its robustness, flexibility, and superior solution quality make it a powerful tool for managing HESs cost-effectively and reliably^46^.

### APO Scalability and extension to complex systems

The scalability of the APO is a critical factor in managing larger or more complex hybrid systems, particularly those integrating energy storage, diverse renewable sources, and multi-micro grid configurations. APO’s bio-inspired adaptability allows it to handle real-time fluctuations in renewable generation, such as solar and wind, by dynamically optimizing power distribution. For hybrid systems with energy storage, APO can optimize charge–discharge cycles to improve efficiency, minimize energy losses, and maintain system stability. Factors such as state of charge, battery efficiency, and degradation over time can be adaptively managed, similar to how APO currently addresses fluctuations in solar power. This integration further enhances energy use optimization and overall system reliability^48–50^.

Scaling APO to multi-micro grid systems opens opportunities for optimizing energy exchange between interconnected grids. Its adaptive local search and real-time response capabilities can balance power flows across multiple grids, ensuring efficient supply–demand equilibrium throughout the network. APO’s modular design and low computational cost make it suitable for large-scale systems, as it avoids reliance on centralized control. Instead, it functions independently at each microgrid level, promoting flexibility and resilience in the broader system. However, as system complexity grows, the optimization problem becomes more challenging, and communication overhead between grids can increase, potentially impacting real-time performance^48–50^. While APO is effective for medium-scale systems, hybrid AI models combining methods like genetic algorithms (GA) and neural networks may outperform it in convergence speed for large-scale setups. These models, however, often sacrifice interpretability. To enhance APO’s scalability, future efforts should focus on distributed optimization techniques, advanced forecasting for resource management, and comprehensive system testing under diverse conditions. Integrating parallel computing or hybrid models could further address high-dimensional optimization problems involving multiple objectives, such as cost reduction, storage efficiency, and reliability. These enhancements would enable APO to manage complex, large-scale hybrid energy systems effectively while maintaining its strengths in adaptability and simplicity^48–50^..

## Energy management strategies for hybrid PV/diesel energy systems

**Fig. 5:**
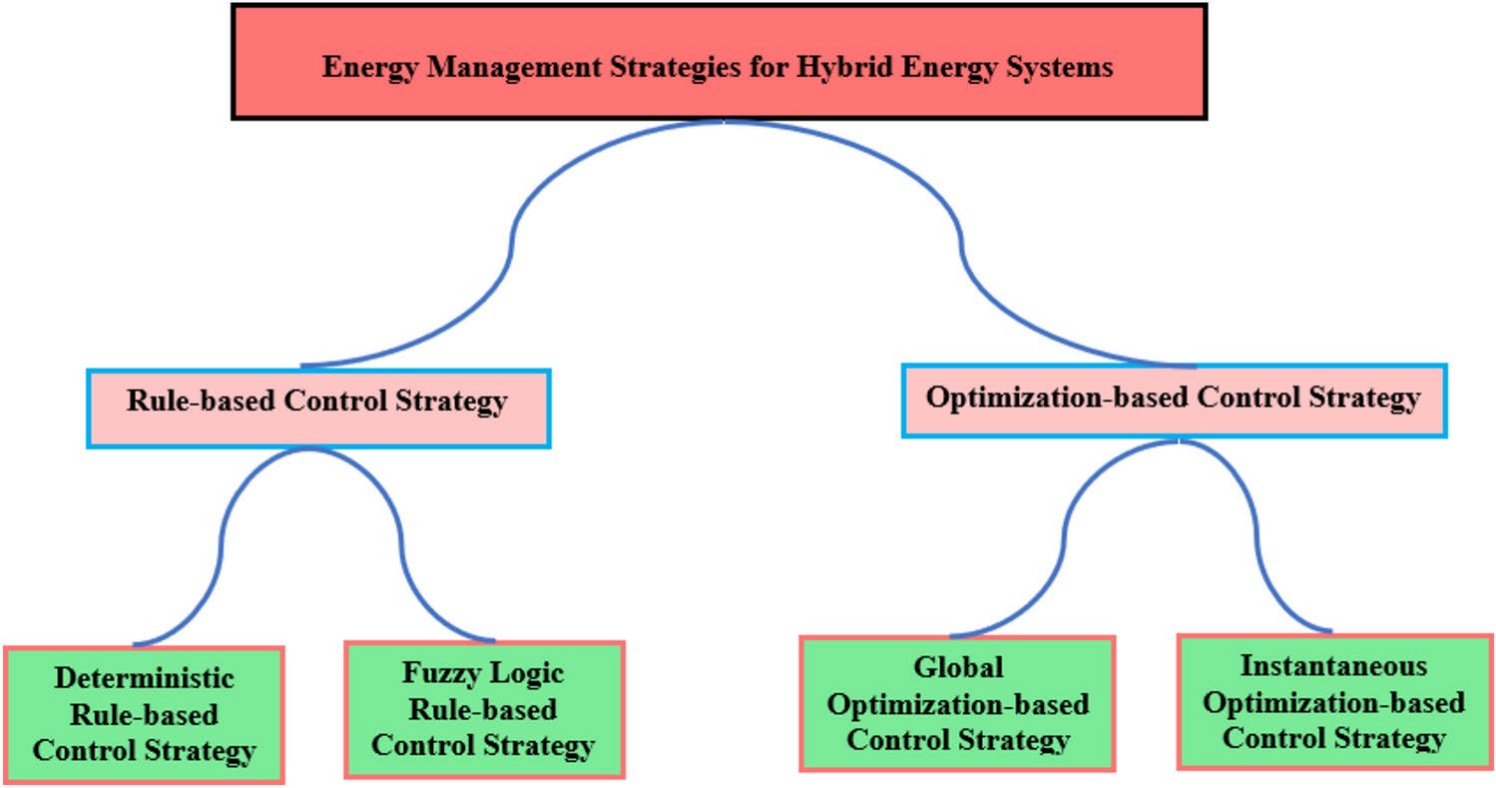
Classification of energy management strategies.

In a PV/D-HS without battery storage, the energy management strategy relies solely on the PV array and DG to meet the load demand. The primary goal is to maximize the use of PV power while ensuring a reliable power supply using the DG as a backup. The hybrid system’s energy management strategies can be categorized based on the topological structure depicted in Fig. 5^51–53^.

In this work, classical and modified energy management strategies have been studied as the following:

### Classical energy management strategy (CEMS) based on inverter ON/OFF control (IOOC)

In this scenario, the inverter ON/OFF control is critical in determining whether the load is supplied by PV, diesel, or both. Since there is no battery storage, the system must carefully balance the real-time generation and consumption. The inverter is turned ON when the power generated by the PV array is sufficient to meet or contribute significantly to the load demand.

**Fig. 6:**
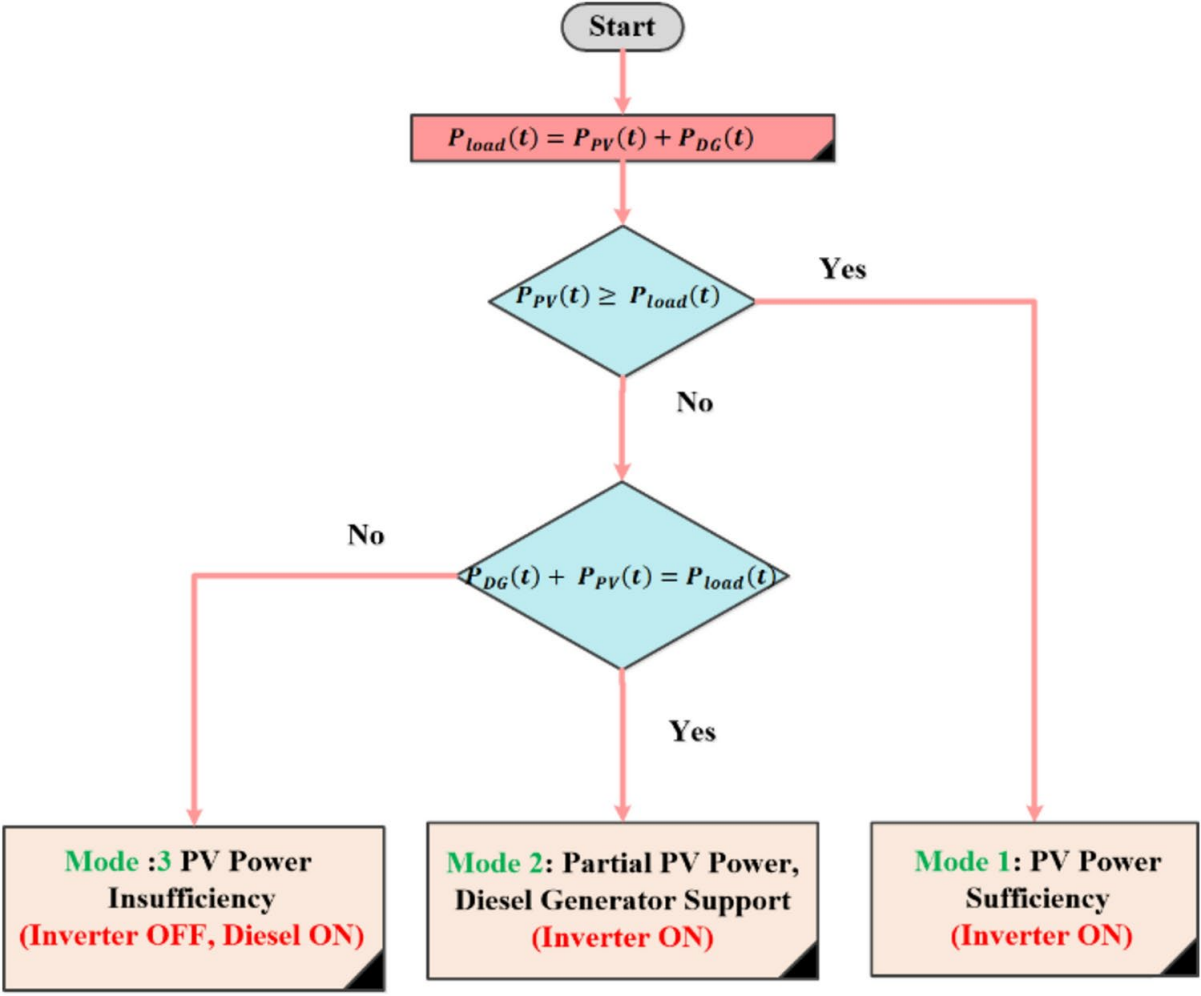
Flowchart of inverter ON/OFF control strategy.

The inverter is turned OFF when the PV power is insufficient to meet the load demand, necessitating complete reliance on the DG. The energy management strategy can be broken down into the following operational modes, as shown in Fig. 6^51–53^:

### Mode 1: PV power sufficiency (Inverter ON)

When the PV array generates enough power to meet the load demand, the inverter is ON, and the PV system supplies the load entirely. The DG remains OFF in this mode, minimizing fuel consumption^54^.

12 $${P}_{PV}\left(t\right) \ge {P}_{load}\left(t\right)$$

### Mode 2: Partial PV power, DG support (Inverter ON)

When PV power is available but insufficient to meet the entire load demand, the inverter remains ON, and the DG is turned ON to supply the deficit. This mode allows the PV system to contribute as much as possible to the load, reducing diesel fuel usage^54^.

13 $${P}_{DG}\left(t\right)+ {P}_{PV}\left(t\right)={P}_{load}\left(t\right)$$

### Mode 3: PV power insufficiency (Inverter OFF, Diesel ON)

When PV power is negligible or absent (e.g., during night time or cloudy conditions), the inverter is turned OFF, and the DG supplies the entire load. In this mode, the DG operates as the primary power source^54^.

14 $${P}_{DG}\left(t\right)={P}_{load}\left(t\right)$$

The inverter ON/OFF control strategy in a PV/D-HS without battery storage has several drawbacks that can affect the overall efficiency and reliability of the system. One major issue is the frequent switching of the inverter, which can lead to increased wear and tear, reducing the inverter’s lifespan and leading to higher maintenance costs. Additionally, this strategy may not fully optimize the use of available PV power, as any excess energy is wasted when the inverter is off, leading to higher reliance on the DG, increased fuel consumption, and greater operational costs. Another significant drawback is the potential for power quality issues or brief interruptions in the power supply when switching between the inverter and the DG, which can cause disruptions or damage to sensitive equipment. Furthermore, the binary nature of the ON/OFF control does not allow for fine control over power output, making it challenging to efficiently manage fluctuating loads or variable PV output. These factors collectively result in suboptimal system performance, reduced energy efficiency, and potential reliability concerns. In this paper, the authors provide a novel strategy to improve the integration of PV energy into the energy system while guaranteeing the dependable operation of the entire system. This strategy aims to overcome the limitations of the inverter ON/OFF management method in a PV/D-HS^54,55^.

### Modified energy management strategy (MEMS) based on Inverter Active Power Control (IAPC) using FO-PID and APO

This work introduces an advanced approach to optimizing the performance of hybrid energy systems based on the FO-PID controller and APO algorithm. FO-PID-based APO is designed to dynamically adjust the inverter’s active power output to maximize the use of solar energy, minimize fuel consumption, and ensure a reliable power supply. The primary objective of the MEMS using FO-PID and APO is to reduce the total operational cost, which includes fuel consumption and maintenance costs of the DG, while maximizing the utilization of PV power. The EMS seeks to balance the power contributions from the PV array and the DG to meet the load demand efficiently.

#### Optimization problem

**Fig. 7:**
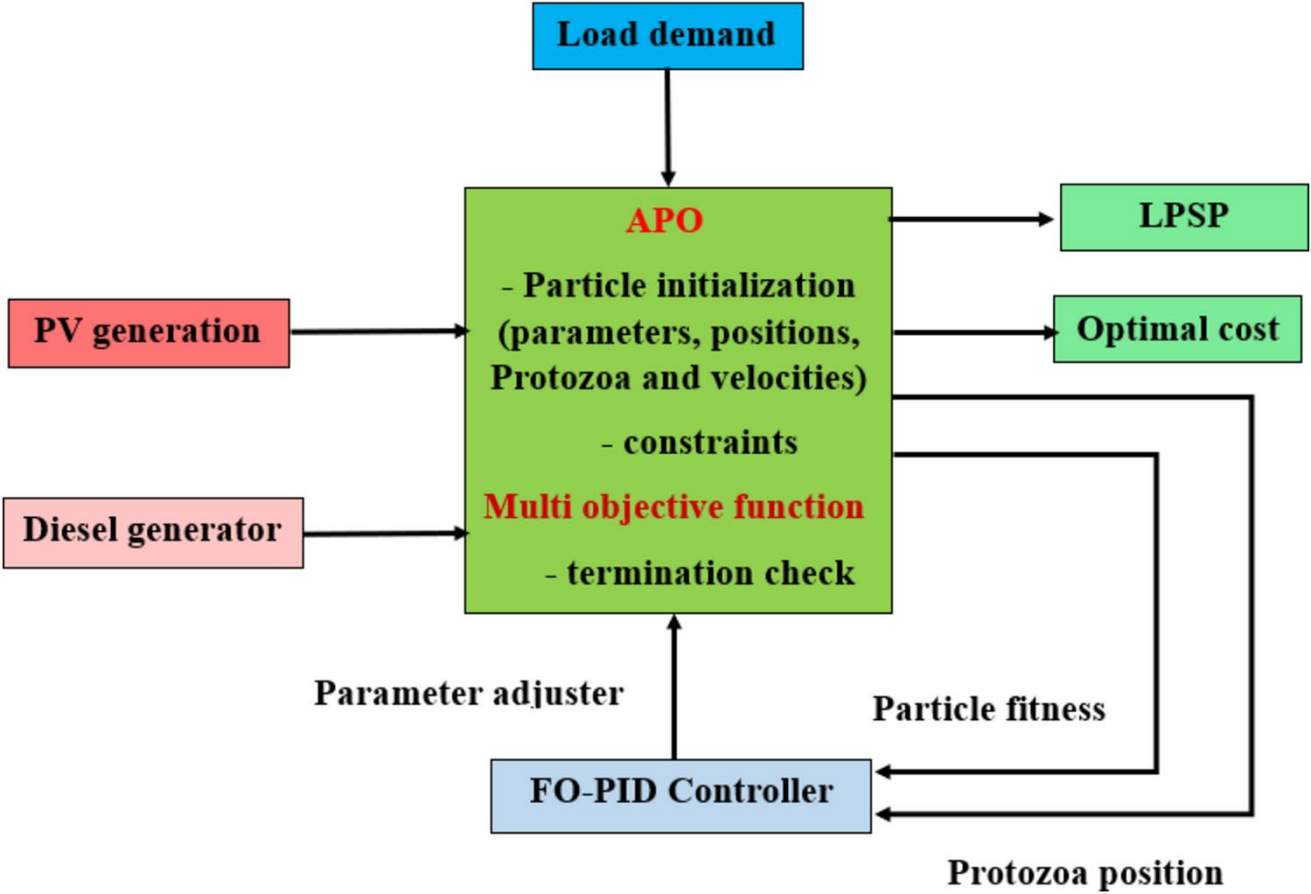
Proposed FO-PID-APO optimization structure.

In this strategy, the FO-PID-APO algorithm with a Multi-Objective Function is employed to optimize multiple conflicting objectives simultaneously, explicitly focusing on minimizing the system’s LPSP and NPC to enhance the efficiency and reliability of energy systems. The core objective of the MEMS is to control the active power output of inverters to balance the supply from renewable sources (such as photovoltaic panels) and conventional sources (like DGs). Figure 7illustrates the architecture of a MEMS-based on an APO algorithm and FO-PID Controller for managing a PV/D-HS^56^.

### Loss of LPSP

The LPSP represents the likelihood that the system will fail to meet the demand. This probability can be minimized by ensuring that the inverter’s active power output is sufficient to cover the demand under various conditions. A lower LPSP means higher reliability, which is crucial for systems in remote areas where power interruptions can have significant consequences. The LPSP is defined as^56–67^:

15 $$LPSP=\frac{\sum {E}_{deficit}}{\sum {E}_{demand}}$$

where: ​${E}_{deficit}$ denotes the total energy shortfall when the hybrid system cannot meet the load demand. ​${E}_{demand}$ denotes the total energy demand over a given period.

Minimizing the LPSP ensures that the system delivers power reliably, even when the PV output fluctuates due to weather conditions or at night.

#### Net present cost (NPC)

The NPC represents the system’s total cost over its lifespan, including initial capital investment, operating expenses, fuel costs for the DG, and maintenance. The goal is to minimize the NPC to make the system economically feasible. The NPC can be expressed as^56–64^:

16 $$NPC={C}_{initial}+\sum_{t=1}^{n}\frac{{C}_{operational}\left(t\right)+{C}_{maintenance}\left(t\right)+{C}_{fuel}\left(t\right)}{{\left(1+T\right)}^{t}}$$

where: ${C}_{initial}$: The initial investment cost (e.g., PV panels, inverter, DG). ${C}_{operational}\left(t\right):$ The operational cost at *t*. ${C}_{maintenance}\left(t\right)$ : The maintenance cost at $t$. ${C}_{fuel}\left(t\right)$: The fuel cost for the DG at *t*. $T$ is the discount rate and $n$ denotes the lifespan of the system.

Reducing the NPC helps optimize the hybrid system’s financial performance, especially in remote or off-grid applications with high fuel and maintenance costs.

#### Multi-objective function

The multi-objective function in our approach simultaneously optimizes three key objectives: minimizing the LPSP to enhance system reliability, minimizing the NPC to ensure long-term financial viability by accounting for the capital, operational, and maintenance costs of the DG and PV system, and maximizing PV utilization to increase the use of renewable energy, thereby reducing operational costs, environmental impact, and dependency on the DG. We employ a weighted sum method to achieve a balanced trade-off among these objectives, assigning weights to each objective based on its relative importance. Prioritizing system reliability involves a higher weight for minimizing LPSP, reducing financial costs emphasizes a higher weight for minimizing NPC, and promoting renewable energy usage requires a higher weight for maximizing PV utilization. Mathematically, the multi-objective function can be represented as^56–67^:

17 $$\text{Multi}-Objective f\left(x \right)= \alpha .LPSP\left(x\right)+\beta .NPC(x)+\upomega .PV Utliation (x)$$

$f\left(x\right)$: is the overall objective function to be minimized. $\alpha$ , β,ω: are weighting factors that balance the importance of $LPSP \left(x\right)$ $NPC(x)$ and $PV Utliation (x)$ $LPSP (x)$ ,$NPC (x)$ and $PV Utliation (x)$ represent the specific function for each objective depending on system configuration.

#### Power balance constraint

The total power supplied by the PV array and the DG must equal the load demand at any given time^56–67^:

18 $${{P}_{inv}\left(t\right)+ P}_{DG}\left(t\right) ={P}_{load}\left(t\right)$$

where ${P}_{inv}\left(t\right)$ denotes the power supplied by the inverter.

#### Inverter power control

The inverter’s output power ${P}_{inv}\left(t\right)$is determined by the APO algorithm, which optimizes the active power based on real-time conditions^56–67^:

19 $${P}_{inv}\left(t\right)={APO (P}_{PV}\left(t\right),{P}_{load}\left(t\right),{P}_{DG}\left(t\right))$$

#### Operational modes

The EMS strategy with APO operates in three primary modes^56–67^:

#### Mode 1: PV dominant mode (Inverter controlled by APO)

In this mode, the APO optimizes the inverter output to meet the load demand primarily with PV power. The algorithm adjusts ${P}_{inv}\left(t\right)$to ensure that the maximum possible PV power is utilized, minimizing DG usage^56–67^.

20 $${P}_{inv}\left(t\right)={APO (P}_{PV}\left(t\right),{P}_{load}\left(t\right))$$

21 $${P}_{DG}\left(t\right)=0$$

22 $${P}_{inv}\left(t\right)\ge {P}_{load}\left(t\right)$$

#### Mode 2: Hybrid mode (APO balances PV and diesel)

When PV power is insufficient, the APO algorithm determines the optimal split between PV power and DG output. It adjusts the inverter to contribute as much PV power as possible, with the DG providing the balance^56–67^.

23 $${P}_{inv}\left(t\right)={APO (P}_{PV}\left(t\right),{P}_{load}\left(t\right)-{P}_{DG}\left(t\right))$$

24 $${P}_{DG}\left(t\right)={P}_{load}\left(t\right)-{P}_{inv}\left(t\right)$$

25 $${P}_{inv}\left(t\right)<{P}_{load}\left(t\right)$$

#### Mode 3: Diesel dominant mode (Minimal PV contribution)

When PV power is negligible, the APO minimizes inverter output and allows the DG to supply the entire load. The algorithm ensures the DG operates efficiently, avoiding inefficient low-load conditions^56–67^.

26 $${P}_{inv}\left(t\right)=\text{APO}(0,{P}_{load}\left(t\right))$$

27 $${P}_{DG}\left(t\right)={P}_{load}\left(t\right)$$

The APO algorithm continuously monitors the system’s parameters, such as PV generation, load demand, and DG efficiency. It adjusts the inverter’s power output according to the following control equation:

#### Constraints

The EMS must satisfy several operational constraints^56–67^:

Inverter capacity

28 $${0\le P}_{inv}\left(t\right)\le {P}_{inv,\text{max}}$$

where ${P}_{inv,\text{max}}$ denotes the maximum output of the inverter.

DG operating limits

29 $${{P}_{DG,min}\le P}_{DG}\left(t\right)\le {P}_{DG,max}$$

where ${P}_{DG,min}$ and ${P}_{DG,max}$are the minimum and maximum operational limits of the DG^56–67^.

The EMS based on modified IAPC using the FO-PID-based APO provides a sophisticated means to manage hybrid PV/Diesel energy systems. By dynamically adjusting the inverter’s output power, the system can efficiently balance the contributions of PV and diesel power to meet load demands, reduce fuel consumption, and lower operational costs. The APO’s ability to optimize real-time power distribution makes it a powerful tool for enhancing the performance and reliability of hybrid energy systems. One of the critical challenges in real-time energy management systems is the response time required to adjust control actions under dynamic conditions. While our current approach focuses on optimizing energy usage and minimizing costs, we recognize the importance of real-time response times for grid stability and efficient operation, particularly in systems with rapidly fluctuating energy sources like solar power^68^. In future work, we plan to investigate the impact of response times on system performance and incorporate mechanisms to ensure the EMS can react promptly to changes in load demand, solar irradiance, and other system variables. This may involve optimizing response times alongside energy allocation, potentially leading to more advanced control algorithms.

Over time, components such as PV panels, inverters, and energy storage systems experience degradation due to wear and tear, environmental factors, and operational cycles. This degradation can impact the efficiency and performance of hybrid energy systems. While our current EMS approach assumes idealized system conditions, we acknowledge the need to incorporate system degradation models into EMS constraints to make the approach more realistic and applicable to real-world scenarios. Future research will explore how degradation affects system performance and develop EMS adaptations to account for it, ensuring optimal operation even as components age. Integrating hybrid systems with the main electrical grid introduces additional complexities, including ensuring compatibility with grid regulations, maintaining power quality, and managing grid frequency and voltage fluctuations. Although our current work focuses on standalone hybrid systems, we recognize the importance of addressing grid-connected operations. Future research will extend the proposed EMS to tackle grid integration challenges. This includes developing strategies for smooth power exchange between the hybrid system and the grid, managing power flows to avoid grid instability, and ensuring compliance with grid codes.

## Result and discussion

### CEMS of PV/D-HS under IOOC

**Fig. 8:**
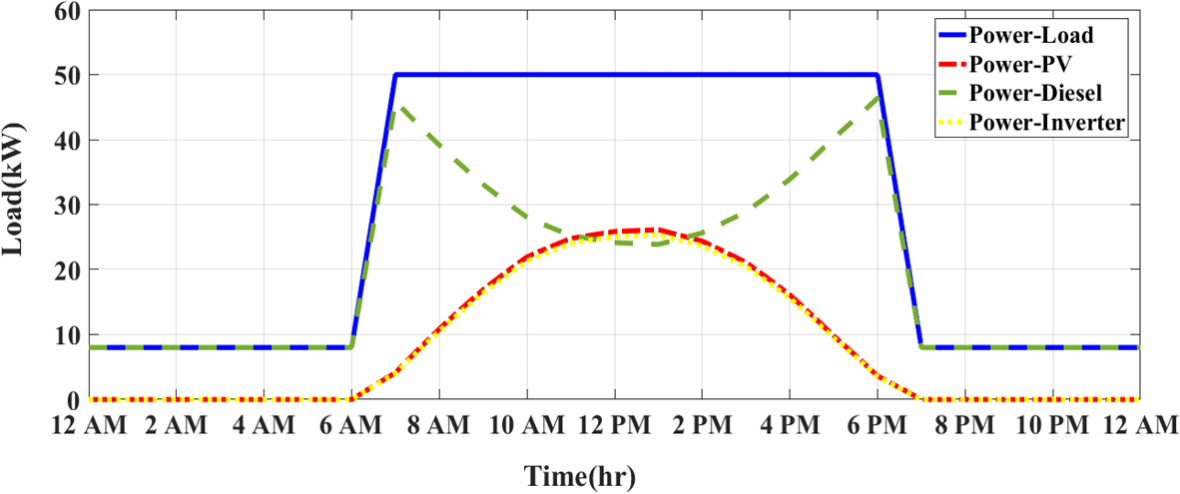
CEMS on a sunny day under IOOC.

Figure 8 presents the energy management system (CEMS) for a sunny day under the integrated optimization and operation control (IOOC) strategy according to realistic waveforms of solar radiation and temperature at Qalyubia City, Egypt, in Fig. 3. The system is modeled during the simulation with a diesel generator (DG) and a photovoltaic (PV) system, utilizing key parameters such as a 50 kW DG and a 30 kW PV array. The system’s operation is simulated using MATLAB/Simulink, with realistic weather data based on historical solar radiation patterns in Qalyubia City. The load demand follows a typical daily consumption curve, starting at a low value during the early hours (12 AM to 7 AM), with the DG operating independently to meet the system’s power needs. The PV system generates electricity as the sun rises around 7 AM, and the load demand increases significantly. From 7 AM to 5 PM, the system experiences peak demand (approximately 50 kW), during which the PV system produces energy in combination with the DG. The DG output is minimal during the midday hours (12 PM to 2 PM) when solar energy production is at its peak. During this period, the PV system takes the lead in energy production, and the DG is only required to supply the remaining power if necessary. Importantly, all PV energy generated is fully utilized, with no curtailment or wastage, as the inverter efficiently consumes the produced energy to meet the load demand.

**Fig. 9:**
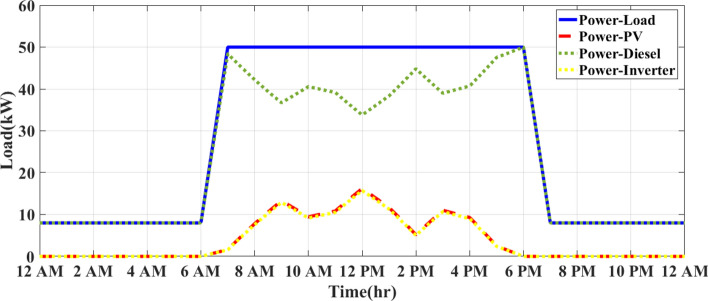
CEMS of PV/D-HS on cloudy days under IOOC.

On a cloudy day, as depicted in Fig. 9, the PV system begins generating power after 7 AM. However, due to fluctuating solar radiation levels, the output is lower compared to the sunny day scenario. The PV system experiences variations in output, peaking at 10 kW to 12 kW. The DG plays a significant role in compensating for the reduced PV power, especially when cloud cover limits the PV output. The DG ensures that the system maintains a reliable power supply despite the variability in solar energy production. The simulations were carried out using a detailed model that incorporates assumptions for solar radiation, DG efficiency, PV system output, and load demand specific to the region. The use of MATLAB/Simulink allowed for real-time control and optimization of the hybrid system using an FO-PID controller, which dynamically adjusts the system to minimize fuel consumption and ensure the efficient use of renewable energy. These simulations demonstrate the effectiveness of the IOOC strategy in optimizing power generation from both renewable and conventional sources.

As shown in Fig. 9, the DG compensates for the reduced solar power by increasing its output to meet the load demand. The DG operates at higher output levels during the day than on a sunny day because the PV system cannot continuously provide full power.

During midday (from 12 to 2 PM), the PV system reaches its maximum output despite the clouds, and the DG is required to supply less power. However, unlike sunny days, the DG remains active because the PV system alone cannot meet the high demand of around 50 kW. Minimal output point for diesel: Unlike on a sunny day, where the DG output drops to minimal levels, the cloudy conditions prevent such a significant reduction. The DG continues to produce higher power levels throughout the day to compensate for the intermittent nature of solar energy.

### MEMS of PV/D-HS under IAPC using FO-PID and APO

**Fig. 10:**
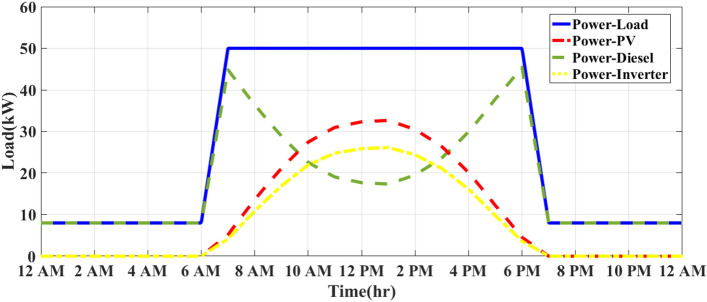
MEMS of PV/D-HS on a sunny day under IAPC using FO-PID and APO.

**Fig. 11:**
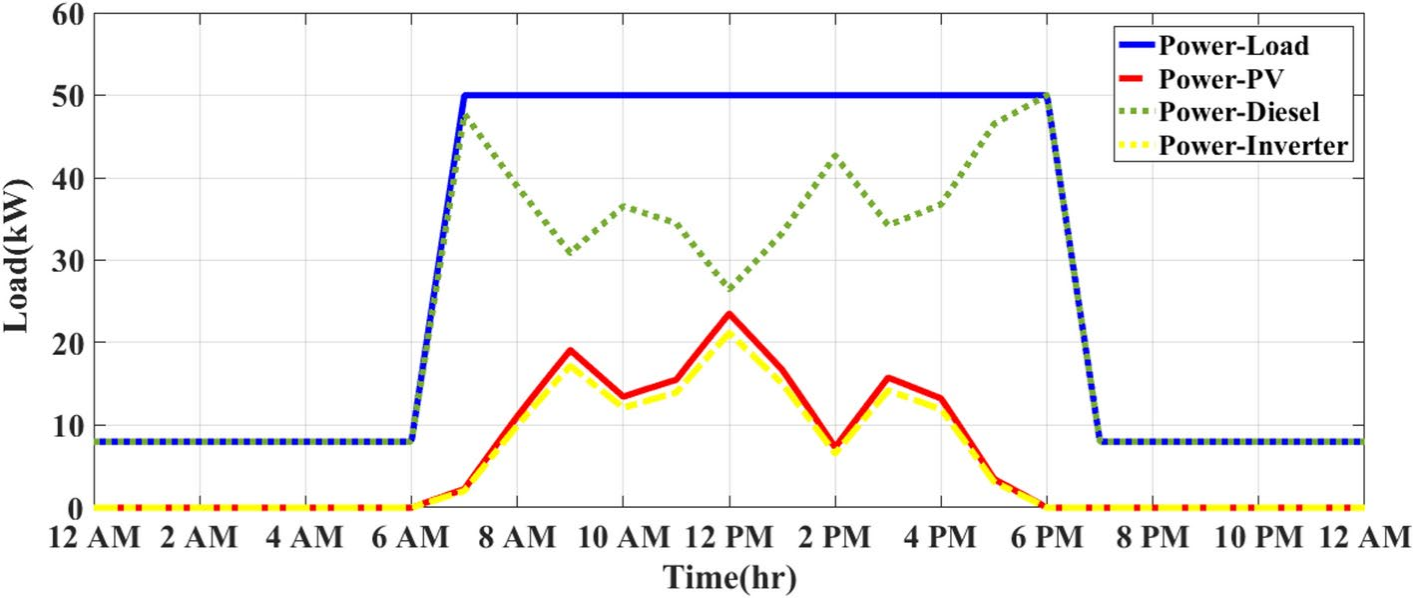
MEMS of PV/D-HS on a cloudy day under IAPC using FO-PID and APO.

During overcast conditions, the energy flow balance for this modified strategy produces outcomes equivalent to those of the inverter ON/OFF control strategy depicted in Fig. 9. Figure 10 illustrates how the system operates on a sunny day using the MEMS of PV/D-HS under IAPC using FO-PID and APO. As shown in Fig. 10, the modified strategy dynamically manages the inverter to convert all available solar power during the day. The DG only provides backup when PV power is insufficient, leading to efficient fuel usage and maximum reliance on renewable energy. In contrast, as shown in Fig. 8, the ON/OFF strategy switches the inverter ON or OFF based on pre-set conditions (such as PV generation thresholds). This simplistic approach can lead to inefficiencies, as the inverter might not fully utilize available PV energy, wasting solar energy if switched OFF and forcing the DG to run longer than necessary. Figure 11 demonstrates the system’s operation on a cloudy day under the MEMS of PV/D-HS using IAPC with FO-PID and APO. On cloudy days, the modified strategy ensures optimal energy management by dynamically balancing PV energy, inverter operation, and DG backup. The system adapts to intermittent solar power availability, prioritizing renewable energy use while minimizing fuel consumption. This highlights the robustness of the modified strategy in handling varying weather conditions, maintaining system efficiency, and maximizing solar energy utilization.

According to Fig. 10, the modified strategy based on FO-PID and APO ensures that all available PV power is utilized, with no curtailment of solar energy. The inverter efficiently balances the PV system and DG, maximizing solar energy use during the day. In contrast, the ON/OFF strategy, if the inverter is OFF during suboptimal solar generation, can lead to significant curtailment of solar energy, resulting in less effective utilization of renewable resources. The DG is utilized only when necessary, specifically during periods of insufficient solar power (early morning, late afternoon, and nighttime). This optimization reduces diesel consumption, allowing the PV system to take priority when sunlight is available. Figure 11 demonstrates the performance of the MEMS of PV/D-HS on a cloudy day under IAPC using FO-PID and APO. The modified strategy dynamically adapts to fluctuating solar power availability on cloudy days, ensuring optimal energy flow. The system minimizes curtailment of PV energy, leveraging every possible opportunity to use renewable energy while relying on the DG as a backup only when PV generation is insufficient. This approach ensures efficient fuel usage even in challenging weather conditions. In comparison, the ON/OFF strategy uses the DG more frequently during cloudy conditions due to its static operation thresholds, which may lead to higher fuel consumption and reduced reliance on renewable energy. However, the modified strategy prioritizes renewable energy, achieving better resource utilization and system efficiency regardless of variability.

**Fig. 12:**
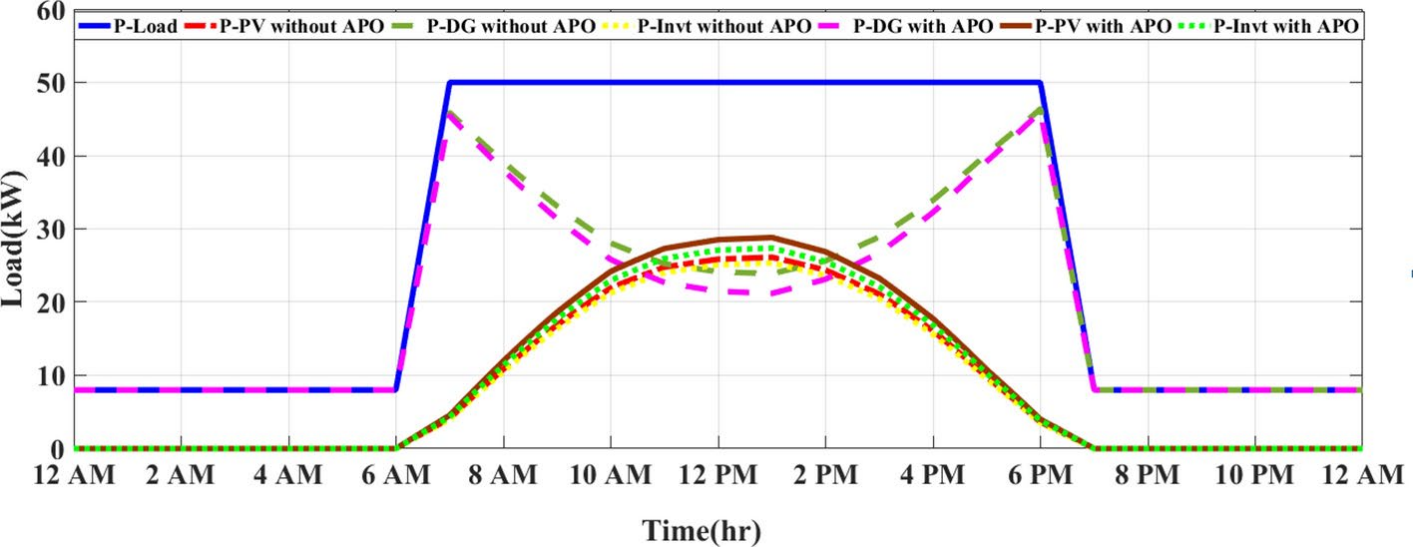
Comparison between CEMS and MEMS of PV/D-HS on a sunny day.

**Fig. 13:**
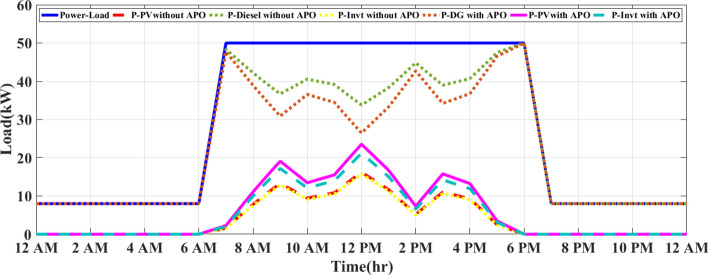
Comparison between CEMS and MEMS of PV/D-HS on a cloudy day.

Figure 12 and Fig. 13 illustrate a comparison between two energy management strategies, MEMS and CEMS in a PV/D-HS on sunny and cloudy days, respectively.

Figure 12 compares the power distribution between MEMS and CEMS in PV/D-HS over a 24-h period. In CEMS, the DG is used more frequently, particularly during high-demand periods and at night when PV power is unavailable. This leads to higher fuel consumption, as the DG compensates for PV’s fluctuating availability without any optimization. Additionally, the inverter operates less efficiently, contributing power in an un-optimized way and failing to balance load demands effectively throughout the day. In contrast, MEMS with APO optimizes PV utilization during daylight hours, reducing reliance on the DG and allowing it to operate at lower capacities or shut off entirely during peak sunlight hours. The inverter in MEMS also responds more dynamically, efficiently distributing power based on available PV generation and demand. This results in a smoother and more sustainable energy flow, as the MEMS system prioritizes renewable energy sources and minimizes fuel consumption by reducing DG operation. Generally, MEMS with APO improves the efficiency of the PV/D-HS by better matching power generation with load demands, reducing costs, and increasing system longevity.

Figure 13 compares CEMS and MEMS of PV/D-HS on a cloudy day. Under cloudy conditions, CEMS relies heavily on the DG due to limited PV generation, exacerbating fuel consumption and reducing system sustainability. The lack of adaptive optimization in CEMS results in an inefficient inverter operation and increased dependency on the DG to meet load demands. In contrast, MEMS with APO demonstrates its robustness even on cloudy days by dynamically adjusting to fluctuating PV availability. The MEMS system prioritizes the efficient use of intermittent solar energy, ensuring minimal curtailment while maintaining optimal inverter and DG performance. By effectively leveraging available PV power, MEMS significantly reduces DG usage, leading to lower fuel consumption and improved energy efficiency. This highlights the superiority of MEMS with APO in managing variable renewable energy sources under diverse weather conditions.

**Fig. 14:**
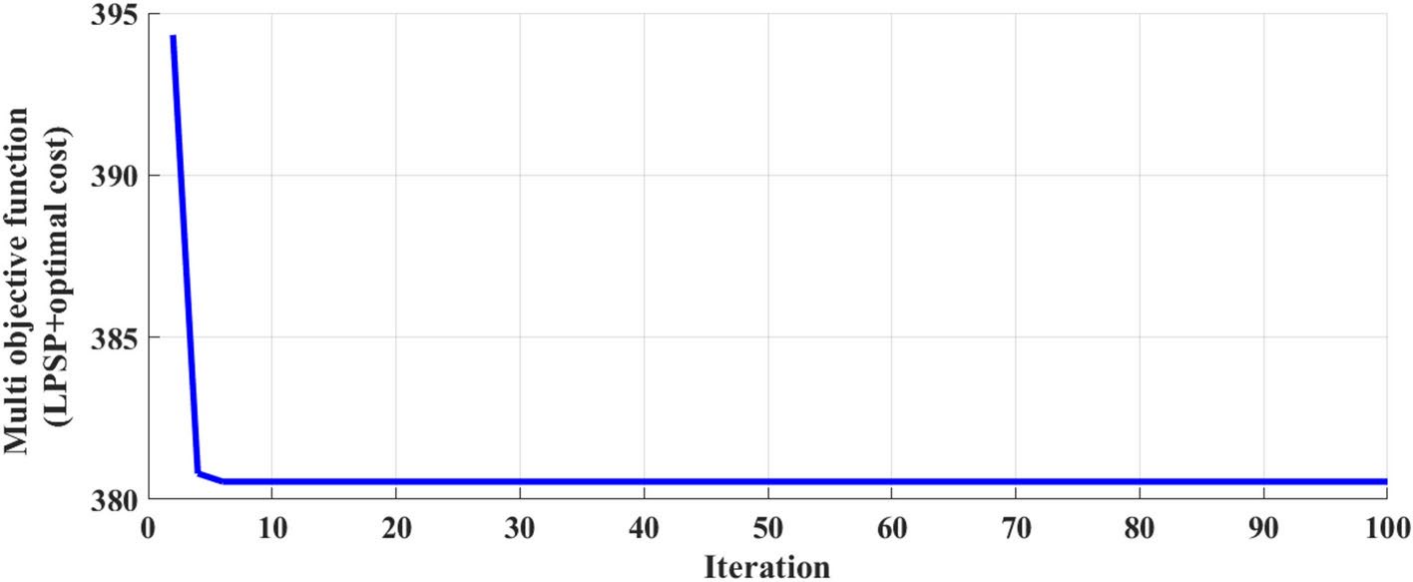
Convergence curve provided by APO for the FO-PID controller.

In this work, the APO is used to determine the optimal gains of the FO-PID controller to achieve better control performance in complex systems such as PV/D-HS. Table 3 provides the optimal gains of the FO-PID controller. Figure 14 presents the convergence curves generated by the APO.

**Table 3:** FOPID controller gains for inverter by APO.

FO-PID Control		
Voltage regulator	*K*_*P*_	19.5375
	*K*_*I*_	891.367
	*lambda*	0.8171
Current regulator	*K*_*P*_	0.723533
	*K*_*I*_	22.1739
	*lambda*	0.88136

The convergence curve shown in Fig. 14 illustrates the optimization process carried out by the APO algorithm for a Multi-Objective Function. This algorithm is designed to address the challenge of simultaneously optimizing multiple conflicting objectives. In this case, the primary objectives targeted by the APO algorithm are the minimization of the LPSP and the NPC of hybrid PV/ diesel energy systems. At the beginning of the optimization process depicted in the curve, the objective function value sharply declines from near 395 at iteration 0 to close to 380 at iteration 7. This rapid drop signifies the algorithm’s ability to efficiently converge towards a solution that effectively reduces LPSP and NPC. By achieving this quick convergence, the APO algorithm demonstrates its capability to simultaneously enhance the efficiency and reliability of hybrid PV/ diesel energy systems by addressing these critical objectives. The curve eventually levels off as the iterations progress, maintaining a relatively constant value. This behavior indicates that the algorithm has reached a plateau where the optimization process has stabilized, resulting in a balanced solution that minimizes both LPSP and NPC. This balanced solution achieved by the APO algorithm showcases its effectiveness in navigating the complexities of multi-objective optimization and successfully optimizing energy systems to enhance efficiency and reliability. The modified strategy involves real-time inverter management to maximize the conversion of DC solar power into AC power. It smooths out fluctuations in solar output, ensuring the load receives a consistent power supply and reducing stress on the DG. Conversely, the inverter in the ON/OFF Strategy operates either at full capacity or not at all, resulting in inefficient power flow and inadequate responses to varying PV system outputs. The modified system responds dynamically to load changes (such as the increase to 50 kW during the day), optimally managing contributions from both the PV system and DG to meet load demands without over-relying on diesel power. In contrast, a simple ON/OFF approach may result in excessive reliance on the DG when some PV power is available, leading to higher operational costs and reduced efficiency.

The inverter active power control strategy using APO offers significant advantages over a basic ON/OFF control strategy. It maximizes the utilization of renewable energy from the PV system, ensuring efficient solar power usage while operating the DG only when necessary. The dynamic control of the inverter facilitates continuous energy flow without wastage or curtailment of solar power, while the APO optimization minimizes fuel consumption, reducing costs and environmental impact. In contrast, the ON/OFF strategy may lead to inefficient energy use, resulting in higher diesel consumption and less effective use of renewable energy.

Table 4 outlines the economic specifications for a hybrid PV/Diesel system, detailing costs for key components. The PV array has an initial capital cost of $2550 per kW, with a low O&M cost of $10 per kW annually and a replacement cost equal to its initial cost, $2550 per kW, over a 25-year lifespan. The PV array has no fuel cost since it generates electricity directly from solar energy. The diesel generator has an initial cost of $100 per kW, with an O&M cost of $0.075 per operating hour and a replacement cost of $100 per kW after 30,000 h of operation. The fuel cost for the diesel generator is estimated at $0.375 per kWh, based on a fuel consumption rate of 0.25 L per kWh and a fuel price of $1.50 per liter. The inverter initially costs $250 per kW, with an O&M cost of $3 per kW annually and a replacement cost of $250 per kW over a 15-year lifespan. No fuel cost is associated with the inverter. This table highlights the cost structure for each system component, emphasizing the renewable PV system’s lack of fuel costs. At the same time, the diesel generator introduces fuel-related expenses, which impact the overall economic performance of the hybrid system.

**Table 4:** Economical Specifications of PV/D-HS.

Component	Initial Capital Cost ($/kW)	O&M Cost ($/kW)	Replacement Cost ($/kW)	Lifetime	Fuel Cost ($/kWh)
PV Array	2560	11	2560	25 years	N/A
Diesel generator	101	0.075/Operating-hr	101	30,000 h	0.375
Inverter	252	4	252	15 years	N/A

**Fig. 15:**
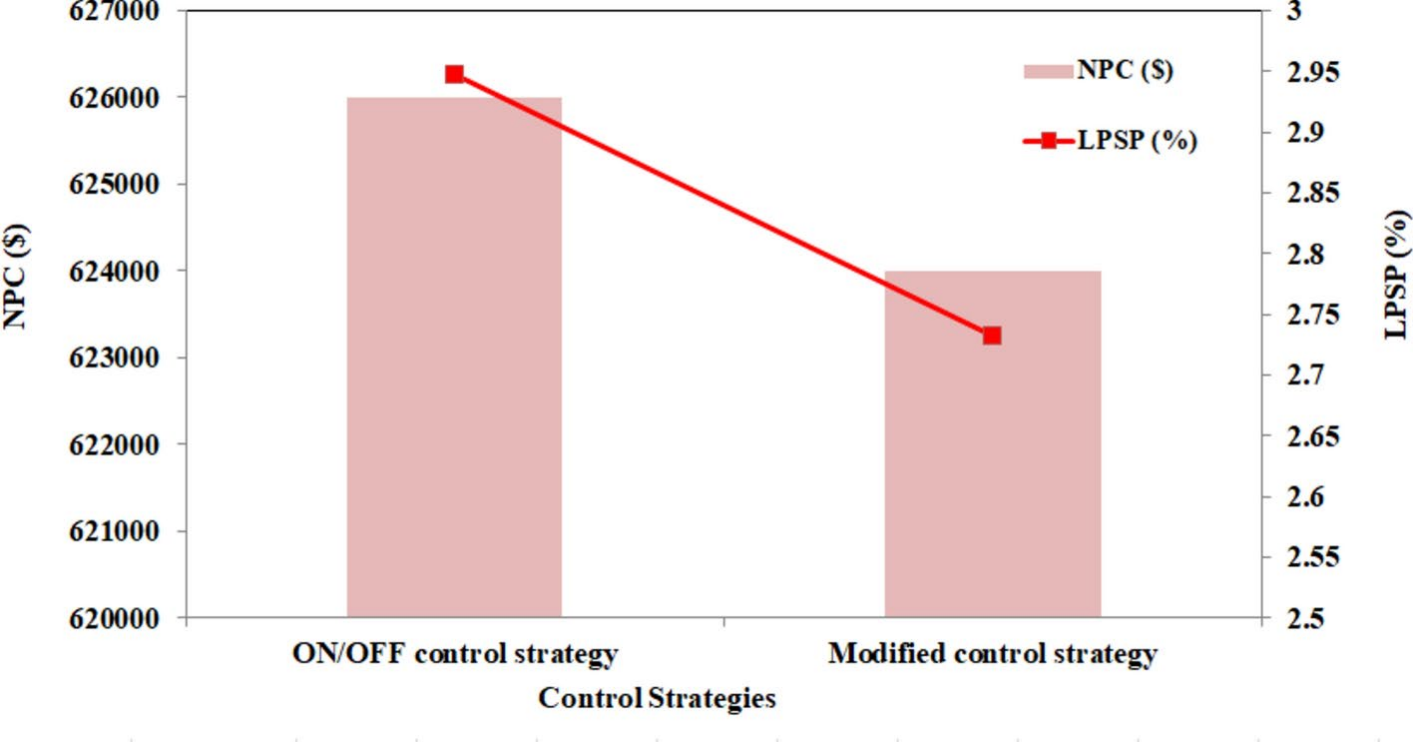
Comparison of LPSP and NPC between the two control strategies.

Figure 15 compares the LPSP and NPC of the two control strategies. The modified control strategy shows lower LPSP and NPC than the ON/OFF control strategy.

### Control strategies’ impact on the solar energy fraction

In a PV/D-HS, control strategies directly influence the Solar Energy Fraction (SEF), the ratio of energy supplied by the solar PV system to the total energy consumed.

30 $$\text{SEF}=\frac{{E}_{PV}}{{E}_{PV}+{E}_{\text{Diesel }}}\times 100$$

**Fig. 16:**
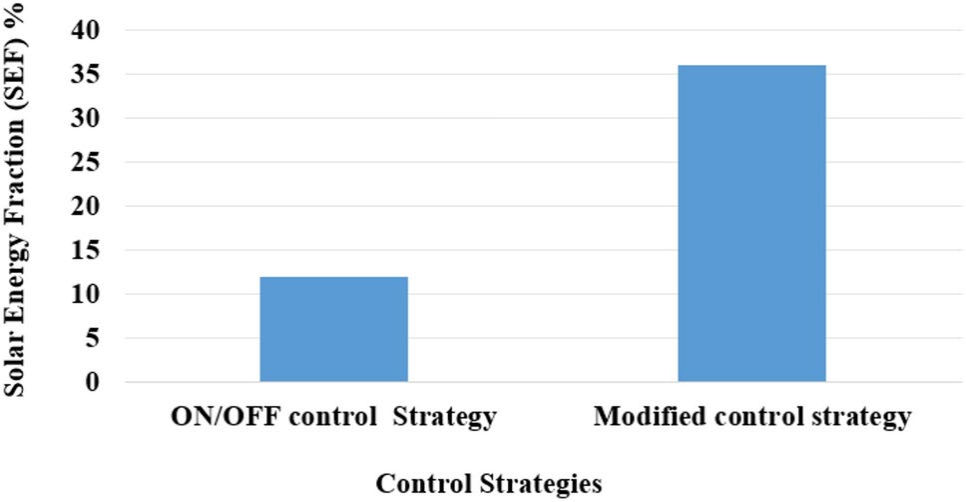
Comparison of control strategies according to SEF on a sunny day.

Figure 16 compares classical and modified energy management according to SEF. The comparison reveals significant differences in performance.

The SEF for the ON/OFF control strategy is notably lower, approximately around 12%, indicating a lower utilization of solar energy. On the other hand, the MEMS achieves a significantly higher SEF of approximately 35% on a sunny day, showcasing a substantial improvement in solar energy utilization efficiency. This comparison underscores the effectiveness of incorporating advanced optimization techniques such as FO-PID and APO in energy management strategies. The MEMS, which integrates FO-PID and APO, demonstrates superior performance in optimizing the SEF. Utilizing these advanced optimization algorithms enables the system to efficiently maximize solar energy resources, leading to enhanced performance and overall energy system efficiency.

### Control strategies’ impact on the level of carbon emissions

**Fig. 17:**
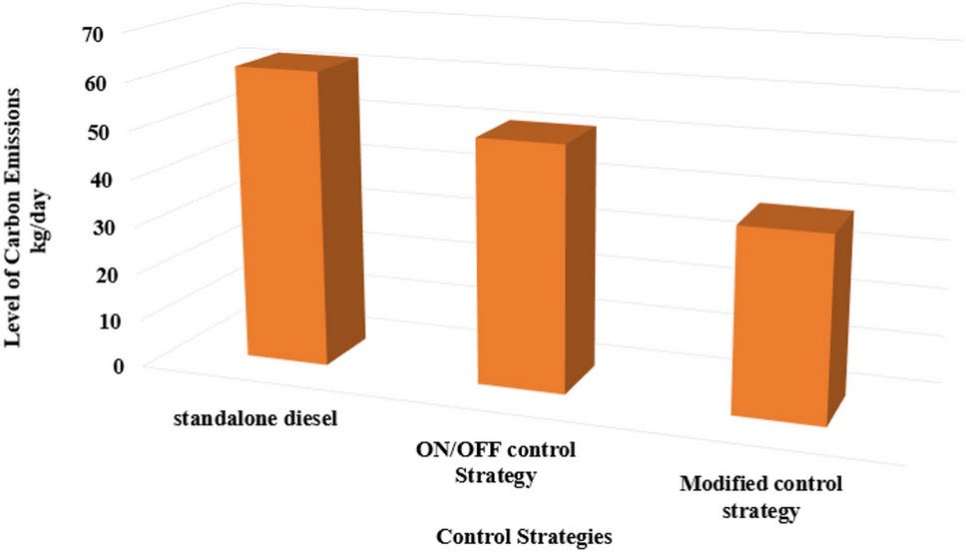
Carbon emission level for different control strategies.

The choice of control strategy in a PV/D-HS significantly affects carbon emissions, especially when comparing a standalone DG with the CEMS and the MEMS, as shown in Fig. 17.

Based on Fig. 17, the comparison of carbon emissions among the standalone diesel generator, the CEMS, and the MEMS reveals a significant trend. The standalone diesel generator emits the highest carbon levels at approximately 62 kg/day, followed by the CEMS at 51 kg/day, while the MEMS achieves the lowest emissions at 38 kg/day. This 24 kg/day reduction compared to the standalone generator represents a 38% decrease in carbon emissions. Such a substantial improvement has broader environmental implications, as it directly contributes to reducing greenhouse gas emissions, a key factor in combating climate change. By lowering emissions, the MEMS enhances operational efficiency and supports global sustainability goals, such as those outlined in the Paris Agreement, promoting cleaner energy use and advancing long-term environmental health.

## Conclusion

This paper presents a MEMS for PV and diesel hybrid systems based on IAPC combined with a FO-PID controller and the APO. The MEMS is designed with a multi-objective function to minimize the LPSP and NPC, encompassing fuel consumption, maintenance costs of the DG, and maximizing PV power utilization. The results show that the MEMS significantly outperforms the CEMS, which relies on simple inverter ON/OFF control. In CEMS, the inverter operates only when PV generation meets or contributes to load demand, leading to periods of complete reliance on the DG. In contrast, the proposed MEMS dynamically adapts to varying environmental conditions and load demands, enhancing system efficiency and reliability. Simulation results demonstrate that the FO-PID-based APO strategy effectively reduces diesel consumption and operational costs while optimizing energy management in hybrid systems. This innovative approach improves economic performance and supports sustainability efforts by decreasing dependence on fossil fuels. The MEMS offers a robust framework for advancing PV/D-HSs, providing valuable insights for future research and practical applications in renewable energy management. For future research, the MEMS could be expanded to larger-scale hybrid systems, which may present new challenges and opportunities for optimization. Additional renewable energy sources, such as wind or hydropower, could further improve system flexibility and energy production. Furthermore, integrating energy storage solutions, such as batteries, would enhance the reliability and efficiency of the system by better-managing fluctuations in renewable energy generation and providing backup during periods of high demand or low renewable output. These areas offer significant potential for improving hybrid energy solutions and advancing sustainability in the energy sector.

## Data Availability

All data generated or analyzed during this study are included in the article.

## Abbreviations

AI: Artificial intelligence
APO: Artificial Protozoa optimizer
CC: Cycle charging
CEMS: Classical energy management strategy
DG: Diesel generator
DP: Dynamic programming
EAs: Evolutionary algorithms
FLC: Fuzzy logic controllers
FO-PID: Fractional-order proportional-integral-derivative
GA: Genetic algorithms
HES: Hybrid energy systems
HRES: Hybrid renewable energy systems
IOOC: Inverter ON/OFF control
IAPC: Inverter active power control
IoT: Internet of things
LF: Load following
LPSP: Loss of power supply probability
LP: Linear programming
MILP: Mixed-integer linear programming
MEMS: Modified energy management strategy
MPC: Model predictive control
NN: Neural networks
NPC: Net present cost
PSO: Particle swarm optimization
PV: Photovoltaic
PV/D-HS: PV/diesel hybrid system
RBCS: Rule-based control strategies
SEF: Solar energy fraction
